# Targeting PKLR and lipogenic enzymes through JNK inhibition to develop a therapeutic strategy for MASLD and MASH

**DOI:** 10.3389/fphar.2026.1823203

**Published:** 2026-07-01

**Authors:** Woonghee Kim, Mengzhen Li, Xinmeng Liao, Sevilay Özmen, Edanur Yıldız, Melik Saraçoğlu, Cem Baba, Fatih Caglar Celikezen, Said Ali Atalay, Ahmet Tuğrul Akkuş, Fatih Alper, Han Jin, Hong Yang, Shazia Iqbal, Jihad Sebhaoui, Sajda Ashraf, Burcu Belmen, Jan Boren, Mathias Uhlen, Cheng Zhang, Hasan Turkez, Adil Mardinoglu

**Affiliations:** 1 Science for Life Laboratory, KTH – Royal Institute of Technology, Stockholm, Sweden; 2 Department of Pathology, Faculty of Medicine, Atatürk University, Erzurum, Türkiye; 3 Institute of Science, Atatürk University, Erzurum, Türkiye; 4 Institute of Science, Erzurum Technical University, Erzurum, Türkiye; 5 Department of Chemistry, Science and Letter Faculty, Bitlis Eren University, Bitlis, Türkiye; 6 Department of Radiology, Faculty of Medicine, Atatürk University, Erzurum, Türkiye; 7 Trustlife Labs Drug Research & Development Center, Istanbul, Türkiye; 8 Life and Health Sciences Laboratory, FMP, Abdelmalek Essaadi University, Tetouan, Morocco; 9 Department of Molecular and Clinical Medicine, University of Gothenburg, Sahlgrenska University Hospital, Gothenburg, Sweden; 10 Department of Medical Biology, Faculty of Medicine, Atatürk University, Erzurum, Türkiye; 11 Centre for Host-Microbiome Interactions, Faculty of Dentistry, Oral & Craniofacial Sciences, King’s College London, London, United Kingdom

**Keywords:** DNL *de novo* lipogenesis, hepatic steatosis, JNK (c-Jun N-terminal kinase), MASLD, new drug

## Abstract

**Background:**

Pyruvate kinase liver and red blood cells (PKLR) is linked to metabolic dysfunction-associated steatotic liver disease (MASLD). Previous study, we identified JNK-IN-5A, a c-Jun N-terminal kinase (JNK) inhibitor that suppresses PKL expression in HepG2 cells using computational drug repurposing and screened out four hit JNK-IN-5A derivatives (SET-151, SET-152, SET-162, SET-130).

**Materials and Methods:**

We validated therapeutic efficacy of JNK-IN-5A and four derivative (SET-151, SET-152, SET-162, SET-130). HepG2 *de novo* lipogenesis (DNL) steatosis model was used *in vitro* validation. RNA sequencing data were analysed using systems biology approaches, including transcriptomic profiling and COMPASS analysis. GLP-like toxicity assessment in rat model shows *in vivo* safety and MASLD rat model revealed *in vivo* therapeutic effect to MASLD and MASH.

**Results:**

In a HepG2 DNL steatosis model, all compounds reduced intracellular triacylglycerol (TAG) and inhibited key DNL proteins (PKL, FASN, ACACA, SCD1, SREBP1-c, ChREBP). Transcriptomic profiling revealed stronger anti-steatotic effects with SET-151, SET-152, and SET-162, which uniquely downregulated genes in pyruvate metabolism, bile acid synthesis, fatty acid metabolism, and glycolysis. Compass analysis showed these derivatives significantly altered lipid-related metabolic reactions, unlike JNK-IN-5A. In a high-sucrose, high-fat diet-induced MASLD rat model, JNK-IN-5A and SET-152 reduced hepatic lipid accumulation, liver stiffness, and MASLD biomarkers.

**Conclusion:**

Our findings identify PKLR as a promising therapeutic target for MASLD and MASH. SET-152 suppressing PKLR through JNK inhibition highlights its potential as a new drug for MASLD and MASH therapy.

## Introduction

Metabolic dysfunction-associated steatotic liver disease (MASLD) is a chronic metabolic liver disease representing a major global health concern, driven by underlying metabolic dysfunction and strongly linked to dysregulated lipid metabolism, obesity and type 2 diabetes (Mardinoglu and Palsson, 2025). Macro vesicular steatosis causes chronic inflammation, which can develop into metabolic dysfunction-associated steatohepatitis (MASH) (Sinton et al., 2021). Chronic hepatic inflammation leads to collagen deposition and fibrotic tissue formation, ultimately progressing to cirrhosis (Dham et al., 2019). Approximately 20% of MASH patients progress to cirrhosis (Sheka et al., 2020). The estimated global prevalence of MASLD among adults is approximately 32%, with a higher prevalence observed in males (40%) compared to females (26%) (Riazi et al., 2022). Currently, resmetirom is the only FDA-approved therapeutic agent for MASH (FDA approves first MASH drug, 2024). Despite this advancement, there remains an urgent need for the development of additional effective therapies for MASH, and extensive research efforts are ongoing in this field.

The liver, as a central regulator of systemic metabolism, takes up and processes various metabolites circulating in the blood. Free fatty acids (FFAs) entering the liver from the bloodstream are converted into triacylglycerols (TAGs) (Bradbury, 2006). Excess carbohydrates are used in the synthesis of fatty acids through *de novo* lipogenesis (DNL) (Browning and Horton, 2004). High-carbohydrate intake, particularly under conditions of hyperinsulinemia and hyperglycaemia, activates key transcription factors such as SREBP-1c and ChREBP (Sanders and Griffin, 2016). These transcription factors upregulate the expression of critical DNL enzymes, leading to increased fatty acid and TG synthesis from glucose (Canbay et al., 2007). To develop effective therapeutic interventions for steatosis, current research is focused on targeting various key regulators within these pathways.

Kristine G. et al. (Griffett and Burris, 2023) reported the development of TLC-2716, an inverse agonist of the liver X receptor (LXR), which is currently undergoing Phase 1 clinical trials. LXR is a key transcription factor that forms a heterodimer with the retinoid X receptor (RXR) and plays a central role in promoting hepatic lipogenesis and steatosis (Shen et al., 2011). By binding to LXR as an inverse agonist, TLC-2716 suppresses hepatic lipogenesis and fibrosis (Griffett and Burris, 2023; Griffett et al., 2015). Another promising therapeutic approach targets hepatic thyroid hormone receptor β (THR-β). Resmetirom (MGL-3196), a selective THR-β agonist, has been developed to activate lipid catabolism in the liver and has recently received FDA approval following successful Phase 3 trials (FDA approves first MASH drug, 2024; Harrison et al., 2023; Kelly et al., 2014). THR-β, activated by triiodothyronine (T3) thyroid hormone, regulates the expression of genes involved in systemic lipid reduction, increased bile acid synthesis, and enhanced fat oxidation (Kannt et al., 2021). Resmetirom has demonstrated therapeutic efficacy in reducing hepatic fat accumulation by selectively activating THR-β (Kannt et al., 2021; Hatziagelaki et al., 2022; Zhou et al., 2022; Karim et al., 2023).

Lee et al. (Lee et al., 2017) applied a systems biology approach to compare healthy liver tissue with samples from individuals with MASLD and hepatocellular carcinoma (HCC). This study identified key genes that were highly upregulated in both MASLD and HCC, highlighting their potential as therapeutic targets. Among these, three genes, including pyruvate kinase liver (PKL) and red blood cell (PKLR), patatin-like phospholipase domain-containing 3 (PNPLA3), and proprotein convertase subtilisin/kexin type 9 (PCSK9), were identified as effective drug targets. PKLR, a key enzyme in the DNL pathway and lipid metabolism, plays a critical role in hepatic steatosis (Liu et al., 2019; Yuan et al., 2025). To advance the development of novel therapeutics for MASLD, we established an *in vitro* model of DNL-induced steatosis and performed computational drug repurposing to identify small-molecule regulators of PKLR. In our previous study, we established a HepG2 DNL steatosis model using insulin and the LXR agonist T0901317 (Kim et al., 2023). The HepG2 cell line is a well-validated and widely used *in vitro* model for studying hepatic metabolism include glucose (Abruscato et al., 2025; Pittala et al., 2024), insulin (Sefried et al., 2018), and lipid metabolism (Lin et al., 2025; Rida and Kreydiyyeh, 2023). We induced DNL in HepG2 cells using insulin and T0901317. This method led to significant increases in intracellular TAG accumulation, upregulation of key DNL pathway transcription factors and enzymes, as well as enhanced glycolytic activity and also showed clinical relevancy compared to MAFLD phenotype human cohorts (Kim et al., 2023).

To identify the modulators of PKLR, we applied a systems biology-driven drug repurposing approach and identified JNK-IN-5A as a small molecule that reduces PKL protein levels in HepG2 cells, exhibiting promising anti-steatotic effects in the HepG2 DNL steatosis model (Kim et al., 2023; Zhang et al., 2022). JNK-IN-5A is a potent and selective ATP-competitive inhibitor of the c-Jun N-terminal kinase (JNK) family. It has been reported that there are strong mechanistic links between the JNK signalling pathways and DNL. It has been widely used to modulate JNK pathways in the *in vitro* and *in vivo* models of metabolic disorders, certain types of cancer and neurodegeneration.

In this study, we evaluated the effect of the JNK-IN-5A, a PKLR-suppressing small molecule that suppresses PKLR, using a HepG2 DNL steatosis model. We observed that JNK-IN-5A treatment significantly reduced TAG accumulation by downregulating DNL pathway protein expression in the HepG2 DNL steatosis model. Furthermore, we identified four novel derivatives of JNK-IN-5A, including SET-151, SET-152, SET-162 and SET-130 that exhibited potent inhibitory effects on DNL-induced steatosis *in vitro*. These derivatives effectively decreased TAG accumulation and suppressed DNL pathway activation. For comparative purposes, we also evaluated the impact of the THR-β agonist resmetirom (Harrison et al., 2024) in the HepG2 DNL steatosis model. Additionally, RNA sequencing was performed to investigate the transcriptomic differences between the treatment groups. To further elucidate the mechanisms of action of JNK-IN-5A and its derivatives, we applied systems biology analyses to identify altered pathways and metabolic reactions. These analyses provided insights into how the compounds modulate key metabolic processes relevant to MASLD. Furthermore, we tested the tolerability of SET-152 in GLP-like toxicity study in rats and evaluated the therapeutic potential of SET-152 in an *in vivo* MASLD model induced by a high-sucrose and high-fat diet. Treatment with the compounds significantly improved MASLD-related symptoms, as demonstrated by magnetic resonance imaging (MRI), two-dimensional shear wave elastography (2D-SWE), liver histology, and blood biochemical markers.

## Materials and Methods

### Cell culture and DNL steatosis induction

HepG2 wild-type cells (ATCC, ATCC HB-8065™, USA) were purchased from the genome engineering company Synthego. Cells were maintained with RPMI 1640 (R2405, Sigma-Aldrich) supplemented with 10% fetal bovine serum (FBS, F7524, Sigma-Aldrich, Germany), 1% P/S (P4333, Sigma-Aldrich, Germany) and used within under passage 20.6 × 10^4^ cells HepG2 cells were seeded into a 96-well plate format for assay, and 1 × 10^6^ cells were plated into a 6-well plate for Western blot and image analysis. DMEM high glucose (D0819, Sigma-Aldrich, Germany) with 10% FBS, 1% P/S supplemented with 10 μg/mL insulin (I9278, Sigma-Aldrich, Germany), and 10 µM T0901317 (T2320, Sigma-Aldrich, Germany) was changed to induce DNL steatosis in HepG2 cells. DNL steatosis media and compounds were changed for 1 week, with a 3-day, 2-day, and 2-day cycle.

### Triacylglycerol (TAG), cell viability (MTT) assay, and BODIPY™ staining

TAG levels were quantified using the Triacylglycerol Assay Kit–Quantification (ab65336, Abcam, United Kingdom). HepG2 cells were lysed and incubated with 50 µL of assay buffer containing 2 µL of Cholesterol Esterase/Lipase for 30 min at room temperature to hydrolyse TAGs. The cell lysate was then mixed with an additional 100 µL of assay buffer and centrifuged using a tabletop centrifuge. A 50 µL aliquot of the supernatant was combined with 50 µL of assay buffer containing 2 µL of Triglyceride Enzyme Mix and 2 µL of OxiRed Probe, followed by a 10-min incubation at room temperature. Absorbance was measured at 570 nm using a microplate reader (Hidex Sense Beta Plus, Germany).

Cell viability was assessed using the MTT (M6494, ThermoFisher, USA) according to the manufacturer’s instructions. BODIPY™ 493/503 (D3922, Invitrogen, USA) was used for fluorescent staining of intracellular TAGs. After fixation with 4% formaldehyde, cells were washed with PBS and incubated with 2 µM BODIPY™ 493/503 in PBS for 15 min in the dark. For counterstaining, 100 nM Phalloidin Alexa Fluor™ 594 (A12381, Invitrogen, USA) in PBS was applied following PBS wash.

### Western blot analysis and CETSA

HepG2 cells were lysed with CelLytic M (C2978, Sigma-Aldrich, Germany) buffer. 20µg protein lysate was prepared with 2x Laemmli Sample Buffer (1,610,737, Biorad, USA). SDS PAGE was conducted using Mini-PROTEAN® TGX™ Precast Gels (Bio-Rad) and transferred by Trans-Blot® Turbo™ Transfer System (Bio-Rad, USA). FASN (ab22759, Abcam, United Kingdom), ACACA (NBP2-55439, Novus, USA), ChREBP (ab92809, Abcam, United Kingdom), SREBP-1C (PA1 337, Invitrogen, USA), SCD-1 (ab236868, abcam, United Kingdom), STAT1 (HPA000982, Sweden), JNK1 (ab199380, Abcam, United Kingdom), JNK2 (ab76125, Abcam, United Kingdom), PKL (06653, Sigma-Aldrich, Germany), PKM (4053S, Cell signalling, USA), Tyr-105 p-PKM (3827S, Cell signalling, USA), and GAPDH (ab8245, Abcam, United Kingdom) were blotted as a primary antibody for overnight. Secondary antibodies, Goat Anti-Rabbit HRP (ab205718, Abcam, United Kingdom) and goat anti-mouse IgG-HRP (ab67895, Abcam, United Kingdom) were blotted for 1 hour. The protein band was detected with ImageQuantTMLAS 500 (29-0050-63, GE, USA). For CETSA, one million HepG2 cells were treated with 20 µM compounds for 2 h s. Cells were washed and prepared into 100 µL PBS in 1.5 mL Eppendorf tube. Heat shock was given by MultiTherm Shaker (H5000-HC, Benchmark scientific) at 50 °C for 3min 300rpm. Proteins were extracted with freeze thaw method using liquid nitrogen. After three times freeze and thaw, samples were centrifuged at 4 °C for 20min max speed.

### Library preparation and RNA-sequencing

The Illumina Stranded Total RNA Prep, Ligation with Ribo-Zero Plus kit was used for the construction of NGS libraries. RNA samples were sequenced with 2 × 100 paired-end reads by the NovaSeq 6,000 system. Raw sequencing data (.bcl) was converted to FastQ with DRAGEN Software (v3.9.5). The data was delivered in FASTQ format using Illumina 1.8 quality scores.

### RNA-seq data pre-processing

The fastq files were first explored by FastQC (v0.11.9) for quality control. Gene expression count data was quantified using the standard protocol of Kallisto (v0.48.0). (Bray et al., 2016). We retrieve the reference cDNA (was GRCh38, Ensembl release 110 for *Homo sapiens*) for alignment and quantification from the Ensembl website. After filtering out non-protein-coding genes and genes with an average count of less than 5, the Kallisto data was used for the downstream analysis. There were 14,444 genes for the downstream analysis.

### Open mechanism of action (MoA)

Open MoA (Liao et al., 2023) was used to predict the potential MoA for compounds. Open MoA integrated network was used as the reference network. Genes with TPM values more than 1.00 were used to build the HepG2 network. MAPK9 (JNK2) was set as the starting point and FDR values of DEGs were computed to construct the weighted network. Eventually, specific weighted subnetworks were built for each of the drugs. In terms of the MoA prediction, ‘most possible path’ function was used to identify the most potential MoA between JNK2-SREBP1-c and JNK2-ChREBP, respectively.

### Differential expression analysis

DESeq2 R package (v1.36.0) (Love et al., 2014) was used to identify the differentially expressed genes (DEGs). To better visualize the data, we adapted *apeglm* method for effect size shrinkage (Zhu et al., 2019). Adjusted P-value <0.05 was chosen as the threshold for the significance of DEGs, with log_2_ fold change >1 for upregulated genes and log_2_ fold change < −1 for downregulated genes. The Benjamini–Hochberg (BH) correction was used for multiple testing corrections. Jaccard index was used to assess the similarity among the DEGs across various treatment groups, which is defined as the size of the intersection divided by the size of the union of two gene sets.

### Principal component analysis

Gene expression profiles after variance stabilizing transformation were used in Principal Component Analysis (PCA) to explore the sample distribution using the R package of pcaMethods (v1.92.0) (Stacklies et al., 2007).

### Gene set functional analysis

Gene set overrepresentation analysis (GSOA) was applied to determine whether known biological functions or processes were overrepresented in the DEGs induced by different treatments. Up and downregulated genes of different treatment groups were extracted for genes of interest and all detected genes were extracted as the background genes. Then, GSOA was applied to determine whether a list of DEGs of interest was significantly associated with specific Gene Ontology (GO) biological process terms.

We also performed gene set enrichment analysis (GSEA) (Subramanian et al., 2005). For this, genes were sorted by log_2_ fold change in descending order, and disease-related genes from DisGeNET (Piñero et al., 2015) were tested for their significance. The R package clusterProfiler (v4.4.4) (Wu et al., 2021; Yu et al., 2012) was used for both GSOA and GSEA.

### Metabolic activity analysis

We used Compass, a flux balance-based algorithm for metabolic model analysis (Wagner et al., 2021). Gene expression levels of all the samples were used as input. The model was created using Recon3D, which was downloaded from the BiGG Models platform (King et al., 2016).

### GLP-like toxicity study in rats

A 7-day oral toxicity study was conducted in Wistar rats (supplier: Envigo, Venray, Netherlands) to preliminarily assess the tolerability of SET-152 at dose levels of 30, 100, and 300 mg/kg body weight. The study included a vehicle control group (4 males and four females) and three SET-152-treated groups, each consisting of three male and three female rats. The vehicle consisted of 1.5% (w/w) hydroxypropyl methylcellulose (HPMC) and 1.5% (w/w) polysorbate 80 (PS80) in 10 mM phosphate-buffered saline (PBS, pH 7), administered at a dose volume of 5 mL/kg. Dosing was performed once daily in the morning for seven consecutive days.

0.5 mL of blood was collected into K_2_EDTA tubes per animal for haematology analysis, and 0.6 mL of blood was collected into lithium heparin tubes for plasma chemistry analysis. All samples were analysed within 60 min of collection. The Exigo haematology analyser was calibrated against a reference sample provided by the manufacturer, and a complete control sample analysis cycle was performed before analysis. All animal procedures and ethical reviews were performed in accordance with the 2010/63/EU Directive on the protection of animals used for biomedical research.

### Animal management and MASH induction *in vivo*

Sprague Dawley rats (age: 6–8 weeks; weight: 250 ± 17 g) were obtained from Experimental Research and Application Center of Atatürk University (ATADEM) with ethical approval. A total of four groups were established. The control group consisted of eight rats and continued to receive standard feed used under normal conditions throughout the study (Bayramoğlu Yem, Erzurum, TURKIYE). The remaining three groups, in which the MASH model was to be induced, were fed a specialized diet (MD.88137), the composition of high sucrose (34% by weight), high fat (21% by weight; 42% kcal from fat), cholesterol (0.2% total cholesterol). All groups had access to clean drinking water (Doyum Su, Erzurum, TURKIYE). The detailed composition of the diet is provided in Supplementary Figure S3. All experimental groups were weighed weekly.

### 2D shear wave elastography and magnetic resonance imaging (MRI)

Ultrasonography and 2D Shear Wave Elastography (2D-SWE) imaging were performed using the EPIQ Elite (Philips, Amsterdam, the Netherlands) device by two well-experienced radiologists. Five measurements with a 1 mm ROI diameter were taken from the liver with a high-frequency eL18-4 linear probe. The average stiffness values in kilopascals (kPa) of these five measurements were recorded. For the MRI measurement, animals were anesthetized intraperitoneally while lying supine with their hind limbs extended parallel to their body. Magnetic resonance imaging (MRI) was performed using a 3 T clinical scanner (Magnetom Skyra; Siemens Healthineers, Erlangen, Germany), with the rats positioned in a prone position. T1 FL2D (Fast Low-Angle Shot Two-dimensional) sequences were used to obtain in-phase and out-of-phase images for evaluating and calculating fat fraction. The T1 FL2D sequence parameters included a TR (repetition time) of 110 m, TE (echo time) of 1.40 m, voxel size of 1.1 × 1.1 × 3.5 mm, 30 slices with a slice thickness of 3.5 mm. Manual measurements were conducted to calculate fat fraction. Signal intensities were detected with a 0.11 mm^2^ ROI diameter from liver and with a 0.3–0.5 mm^2^ ROI diameter from spleen, both on in-phase and out-of-phase images. Fat percentage was measured by 100 × (liver SIIP/spleen SIIP - liver SIOP/spleen SIOP)/(2 × liver SIIP/spleen SIIP).

### Histopathological analysis

At the end of the experiment, tissue samples were fixed in 10% neutral buffered formaldehyde for 48 h and processed using standard histological techniques. Following paraffin embedding, 4 µm-thick sections were prepared and stained with hematoxylin and eosin (H&E) for histopathological evaluation under a light microscope (Nikon Eclipse Ci). Histopathological assessment was performed based on characteristic morphological features. The severity of hepatic steatosis was graded separately, considering both histological activity and fibrosis stage. The activity level was assessed using the MAFLD Activity Score (NAS), calculated as the sum of three histological components—steatosis (0–3), lobular inflammation (0–3), and hepatocellular ballooning (0–2)—yielding a total score ranging from 0 to 8, as described by Kleiner et al. (Kleiner et al., 2005).

### Micronucleus assay in bone marrow smears

Femur bones were isolated from sacrificed rats, and surrounding muscle tissue was removed. Bone marrow was flushed using a sterile syringe with 0.5 mL FBS and 0.5 mL DMEM into Falcon tubes. The suspension was centrifuged at 2000 rpm for 5 min, and the supernatant was discarded. The pellet was resuspended in a drop of FBS, homogenized, and smeared onto slides. After air-drying, slides were fixed in absolute methanol for 10 min. Staining was performed using a commercial kit (ChemBio Laboratory Research, Turkiye) following the manufacturer’s instructions. Slides were examined under a light microscope (100× oil immersion), and 1,000 polychromatic erythrocytes (PCEs) per slide were scored to determine the frequency of micronuclei (MN).

### Statistical analysis for RNA-seq and experimental data

Error bars in the graphs are presented as mean ± standard deviation (SD) for cell-based experiments. Box plots display the median and interquartile range (IQR) for hematology and plasma chemistry measurements. For RNA-seq data, P-values were adjusted using the Benjamini–Hochberg procedure where applicable. Statistical significance was defined as P < 0.05 (or adjusted P < 0.05) and is indicated by an asterisk (*).

## Results

### JNK-IN-5A derivatives effectively inhibit the DNL pathway in a HepG2 steatosis model

**FIGURE 1:**
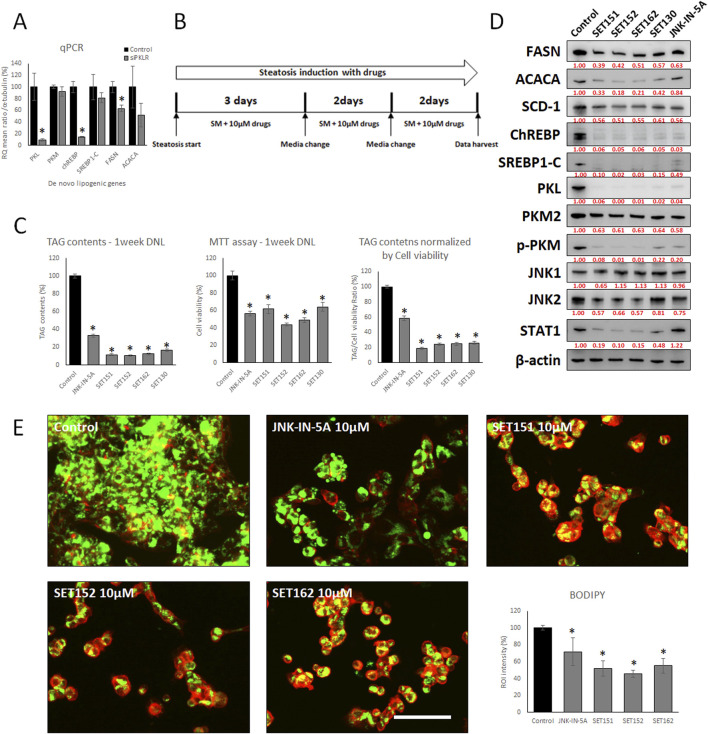
JNK-IN-5A derivatives decrease TAG levels in MASLD cell models. **(A)** siRNA inhibition of PKL expression suppresses the DNL pathway. qPCR were performed with triplicated RNA samples. **(B)** Scheme of HepG2 DNL steatosis model induced by compound treatment. HepG2 cells underwent 1 week of DNL steatosis induction and were treated with 10 µM compounds. **(C)** TAG contents assay, Cell viability assay, and TAG contents normalized by Cell viability assay. Accumulated TAG on 1-week DNL steatosis and 10 µM compounds-treated cells were investigated. Assay was performed as triplicated. **(D)** Western blot analysis for the DNL pathway involved transcription factors and enzymes. Band intensity is measured using ImageJ. **(E)** Fluorescence staining of lipid droplets and the cytoskeleton. Green: neutral lipids, Red: Cytoskeleton (F-actin), Scale bar, 100 μm. ROI of GFP intensity is measured with Image J. Data are represented as mean±SD. Significance was tested using t-test (*P < 0.05).

A systems biology approach previously identified PKL as one of the genes strongly associated with MASLD and HCC (Lee et al., 2017). To explore the functional role of PKL in hepatic steatosis *via* DNL, we examined the expression of key genes involved in the DNL pathway following siRNA-mediated PKL knockdown in the HepG2 *in vitro* steatosis model (Figures 1A,B). PKL knockdown significantly reduced the expression of PKL (9.5%), ChREBP (14.4%), FASN (63%). While not statistically significant, the level of ACACA trended downward by approximately 51.6%.

Building on these findings, we identified JNK-IN-5A as a small molecule that reduces PKL protein levels in HepG2 cells (Kim et al., 2023; Zhang et al., 2022). To enhance therapeutic efficacy, we synthesized a series of JNK-IN-5A derivatives and performed screening at HepG2 DNL steatosis model with 10 µM (Iqbal et al., 2024). We identified four compounds (SET-151, SET-152, SET-162, and SET-130) with more potent anti-steatotic activity. We evaluated these compounds in the 1-week HepG2 DNL steatosis model (Figure 1B). Treatment with 10 μM of each compound significantly reduced TAG accumulation (32.9% for JNK-IN-5A, 11.5% for SET-151, 10.7% for SET-152, 12.3% for SET-162, and 16.7% for SET-130). After normalization to cell viability, TAG reductions were 58.4% for JNK-IN-5A, 18.6% for SET-151, 24.5% for SET-152, 25.1% for SET-162, and 26.1% for SET-130 (Figure 1C). Western blot analysis revealed that all compounds significantly decreased the expression of key DNL pathway enzymes, including fatty acid synthase (FASN), acetyl-CoA carboxylase (ACACA), and stearoyl-CoA desaturase 1 (SCD-1), which catalyses monounsaturated fatty acid (MUFA) production and promotes hepatic TAG storage and VLDL secretion (Figure 1D). Additionally, the expression of DNL-regulating transcription factors, ChREBP and SREBP-1c, was markedly reduced in all treatment groups.

Ito et al. (2013) suggest that JNK2 plays a dominant role over JNK1 in regulating SREBP-1c activation. Consistent with this, JNK-IN-5A and its derivatives did not alter JNK1 protein expression, except for a modest reduction by SET-151 (0.65-fold). Interestingly, SET-151 (0.57-fold), SET-152 (0.66-fold), and SET-162 (0.57-fold) exhibited stronger inhibitory effects on JNK2 expression compared to SET-130 (0.81-fold) and JNK-IN-5A (0.75-fold). Liao et al. (2023), introduced Open MoA, a pipeline to reveal the mechanism of action (MOA) based on network topology and hierarchy. The potential pathway of how JNK-IN-5A regulate PKLR expression was revealed in their study. We employed the Open MoA here to predict two MoAs for all compounds: the ‘JNK2–JUN–SREBP1-c' and ‘JNK2–JUN–MXI1–ChREBP’ signalling pathways (Supplementary Figure S2). Notably, SET-151, SET-152, and SET-162 demonstrated lower penalty scores (1-confidence score) for the MXI1–ChREBP association than SET-130 and JNK-IN-5A, consistent with stronger downregulation of MLXIPL, the gene encoding ChREBP. Similarly, SREBF1, which encodes SREBP-1c, was significantly downregulated following treatment with all compounds, with high-confidence predictions for all interactions within the MoA network. Signal transducer and activator of transcription 1 (STAT1), a transcription factor implicated in the development of MASLD and MASH (Grohmann et al., 2018), is part of the JNK–STAT1–PKL regulatory axis (Liao et al., 2023). We also observed interaction between compounds JNK2 using cellular thermal shift assay (CETSA). We give gradient heat shock to 20 µM compounds treated HepG2 cell lysate. SET151, SET152, and SET162 treated cell lysate showed statistical significant heat resistance at 49.5 °C for 5 min (Supplementary Figure S2C). To check cellular permeability and binding affinity between compounds and JNK2, we treated 20 µM compounds to HepG2 live cells for 2h and give heat shock 50 °C. SET151 (1.47 times), SET152 (1.4 times), and SET162 (1.37 times) showed statistical significant heat shock resistance again (Supplementary Figure S2D). Our data showed that while JNK-IN-5A and its derivatives robustly reduced JNK2 and PKL expression, only the derivatives interact with JNK2 and substantially decreased STAT1 protein levels, indicating enhanced efficacy over JNK-IN-5A.

Increased glycolysis is a characteristic metabolic change in liver steatosis (Lu et al., 2021). Nuclear-localized PKM2, particularly in its monomeric or dimeric form, promotes glycolysis (Yang and Lu, 2013). Phosphorylation at Tyr105 prevents PKM2 tetramer formation, enhancing its glycolytic activity (Hitosugi et al., 2009). While JNK-IN-5A and its derivatives did not affect total PKM2 protein levels in the HepG2 DNL steatosis model, Tyr105-phosphorylated PKM2 levels were dramatically reduced following treatment with all compounds.

Based on these findings, we selected SET-151, SET-152, and SET-162 as the most potent derivatives, demonstrating superior therapeutic efficacy and distinct mechanisms compared to the reference compound JNK-IN-5A (Figure 1E). Their anti-steatotic effects were confirmed by BODIPY™ 493/503 staining for neutral lipids. In the BODIPY assay, SET-151 (52.1%), SET-152 (45.7%), and SET-162 (55.2%) significantly reduced neutral lipid accumulation compared to JNK-IN-5A (71.7%) (Figure 1E). Together, these results demonstrate that SET-151, SET-152 and SET-162 exhibit stronger anti-steatotic activity than JNK-IN-5A, primarily through the inhibition of the DNL pathway and the regulation of key metabolic and transcriptional pathways involved in MASLD progression.

### Comparison of resmetirom and JNK-IN-5A derivatives in the HepG2 DNL steatosis model

**FIGURE 2:**
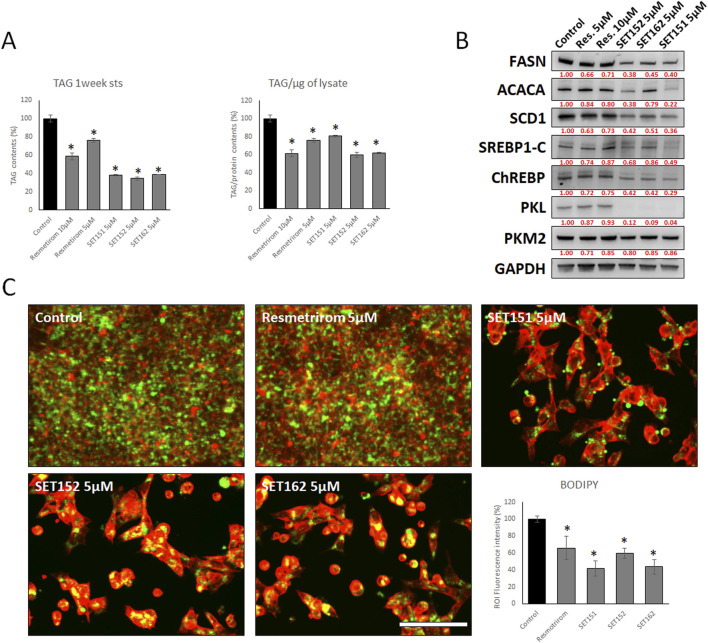
JNK-IN-5A derivatives demonstrated comparable therapeutic efficacy to resmetirom in MASLD cell models. **(A)** TAG contents assay, and TAG contents normalized by protein quantification. Accumulated TAG on 1-week DNL steatosis with resmetirom 10 μM, 5 μM, and 5 µM compound-treated cells was investigated. Assay was performed as triplicated. **(B)** Comparison of resmetirom and JNK-IN-5A derivatives for the DNL pathway, involving transcription factor and enzyme protein expression levels via Western blot analysis. Band intensity is measured using ImageJ. **(C)** 5 µM resmetirom and JNK-IN-5A derivatives lipid droplet and cytoskeleton fluorescence staining. Green: neutral lipids, Red: Cytoskeleton (F-actin), Scale bar, 100 μm. 5 random ROI of GFP intensities are measured with Image J. Data are represented as mean±SD. Significance was tested using t-test (*P < 0.05).

Resmetirom activates hepatic THR-β, promoting systemic lipid clearance by enhancing bile acid synthesis and fat oxidation (Harrison et al., 2023; Harrison et al., 2024). In this study, we compared the therapeutic effects of resmetirom and JNK-IN-5A derivatives in the HepG2 DNL steatosis model (Figure 2). Resmetirom treatment resulted in a dose-dependent reduction in intracellular TAG levels (Figure 2A). Specifically, 10 µM (62.4%) and 5 µM (78.2%) of resmetirom reduced the TAG content. SET-151 (42.8%), SET-152 (38.3%), and SET-162 (43.6%), also significantly reduced TAG content at 5 μM. When normalized to total protein content, 10 µM (61.2%) and 5 µM (75.7%) of resmetirom, 5 µM (80.5%) of SET151, 5 µM (59.9%) of SET152 and 5 µM (61.8%) of SET162 reduced the TAG levels compared to the untreated control group.

Western blot analysis of the DNL pathway proteins further demonstrated the superior inhibitory effects of JNK-IN-5A derivatives compared to resmetirom (Figure 2B). FASN (0.66 & 0.71), ACACA (0.84 & 0.80) and SCD1 (0.63 & 0.73) protein levels were decreased in the 5µM and 10µ resmetirom treated group. In contrast, treatment with 5 μM of SET-151, SET-152, and SET-162 resulted in more pronounced reductions: FASN (0.40, 0.38, 0.45), ACACA (0.22, 0.38, 0.79), and SCD1 (0.36, 0.42, 0.51), respectively.

Moreover, the JNK-IN-5A derivatives showed more potent suppression of the key DNL transcription factors SREBP1-c and ChREBP compared to resmetirom. SREBP1-c expression was reduced by resmetirom at 5 μM (0.74) and 10 μM (0.87), whereas SET-151, SET-152, and SET-162 further decreased SREBP1-c expression (0.49, 0.68, and 0.86). ChREBP expression (0.29, 0.42, and 0.42) was also markedly reduced by JNK-IN-5A derivatives for SET-151, SET-152, and SET-162, compared to reductions observed with resmetirom at 5 μM (0.72) and 10 μM (0.75). Importantly, JNK-IN-5A derivatives almost completely suppressed PKL expression, reducing it to undetectable levels. In contrast, resmetirom-treated cells maintained 87% and 93% of PKL expression at 5 μM and 10 μM, respectively. PKM2 expression was moderately reduced across all treatment groups: resmetirom 5 μM (0.71), resmetirom 10 μM (0.85), SET-151 (0.86), SET-152 (0.80), and SET-162 (0.80).

Lipid staining further confirmed these findings. BODIPY™ 493/503 staining demonstrated that 5 μM resmetirom reduced neutral lipid accumulation to 65.9% of the control level, whereas SET-151, SET-152, and SET-162 showed more pronounced reductions to 41.8%, 59.5%, and 43.6%, respectively (Figure 2C). Collectively, these results indicate that JNK-IN-5A derivatives exhibit stronger inhibitory effects on the DNL steatosis pathway and intracellular lipid accumulation compared to resmetirom at HepG2 DNL *in vitro* model. It highlighting JNK-IN-5A derivatives as a DNL pathway-targeting therapeutics for MASLD.

### JNK-IN-5A derivatives exhibit distinct transcriptional profiles

**FIGURE 3:**
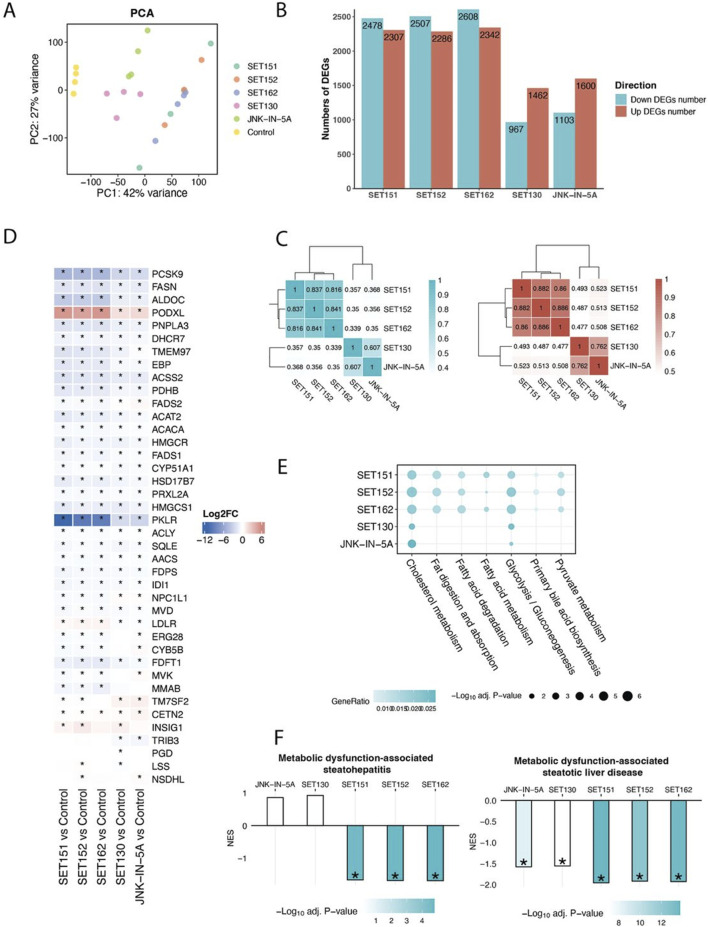
JNK-IN-5A derivatives exhibited distinct transcriptional profiles compared to the reference compound. **(A)** PCA plot showing sample distribution of SET151, SET152, SET162, SET130, JNK-IN-5A, and Control groups (both n = 4) based on principal component analysis of RNA-seq gene expression profile. **(B)** Bar plot showing the numbers of differentially expressed genes (DEGs) in each treatment group. **(C)** Heatmaps of the Jaccard index showing the similarity of up- and downregulated genes across different treatment groups. **(D)** Heatmap showing the differential expression (presented as log2 fold change) of 40 genes derived from the MASLD-associated module in different treatment groups. ∗ Represent significantly differentially expressed genes (DEGs) in each group (cut-off *p* values for DEGs (*p* < 0.05). **(E)** Dot plot showing the GSOA results conducted based on the biological process category from the Gene Ontology (GO) dataset. The size of the dot indicates the gene ratio, i.e., the DEGs assigned to the corresponding pathway relative to the total analysed DEGs., and the dot’s colour indicates the adjusted *p* value. **(F)** Bar plot showing the normalized enrichment score (NES) of metabolic dysfunction–associated steatotic liver disease (MASLD) and metabolic dysfunction–associated steatohepatitis (MASH) related genes. RNA sequencing samples were prepared as quadruple.

To investigate the transcriptional differences induced by JNK-IN-5A and its novel derivatives, we performed global RNA sequencing (RNA-seq) analysis on treated HepG2 cells. Principal Component Analysis (PCA) revealed three distinct gene expression clusters corresponding to the treatment groups (Figure 3A). SET-130 clustered closely with JNK-IN-5A, whereas SET-151, SET-152, and SET-162 formed a separate, tightly grouped cluster. All treatment groups were distinct from the untreated control group.

In terms of global transcriptional impact, SET-151, SET-152, and SET-162 induced substantially higher numbers of differentially expressed genes (DEGs) compared to SET-130 and JNK-IN-5A (Figure 3B). Jaccard index analysis of the DEGs further confirmed that SET-151, SET-152, and SET-162 shared a high degree of similarity in both upregulated and downregulated gene sets (Figure 3C), suggesting a consistent and distinct mechanism of action among these three derivatives. To assess the functional consequences of these transcriptional changes, we first examined the expression of a previously defined MASLD-associated gene module (n = 40) (Yang et al., 2021). All compounds, including JNK-IN-5A and its derivatives, significantly downregulated the expression of genes within this MASLD module (Figure 3D). Notably, SET-151, SET-152, and SET-162 produced a more pronounced downregulation effect, with a marked reduction in PKLR expression.

Gene Set Overrepresentation Analysis (GSOA) revealed that the downregulated genes in all treatment groups were enriched in pathways related to lipid metabolism and glycolysis. Importantly, SET-151, SET-152, and SET-162 uniquely suppressed genes associated with fatty acid metabolism and primary bile acid biosynthesis, pathways highly relevant to MASLD pathogenesis (Figure 3E). To further explore the clinical relevance of these findings, we retrieved MASLD- and MASH-associated genes from the DisGeNET database (773 and 316 genes, respectively), (Piñero et al., 2015), and performed Gene Set Enrichment Analysis (GSEA). The results demonstrated that all treatment groups reduced the enrichment scores of MASLD-associated genes; however, SET-151, SET-152, and SET-162 were significantly more effective in this regard (Figure 3F). Interestingly, a reduction in the enrichment scores of MASH-related genes was observed exclusively in cells treated with SET-151, SET-152, and SET-162, but not in cells treated with JNK-IN-5A or SET-130 (Figure 3F). Collectively, these transcriptomic analyses demonstrate that SET-151, SET-152, and SET-162 exhibit distinct and stronger transcriptional modulation compared to the parental compound JNK-IN-5A, supporting their superior therapeutic potential for MASLD and MASH.

### JNK-IN-5A derivatives exhibit distinct metabolic profiles

**FIGURE 4:**
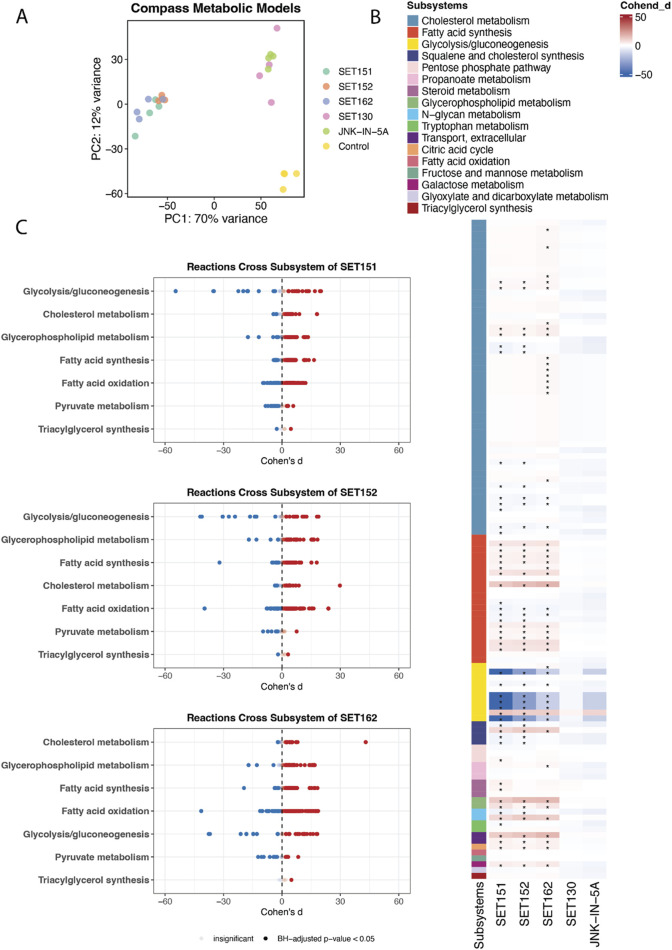
JNK-IN-5A derivatives exhibited distinct metabolic profiles compared to the reference compound. **(A)** PCA plot showing the distinct metabolic profiles among SET151, SET152, and SET172 group, SET130, JNK-IN-5A group and Control group (both n = 4). **(B)** Heatmap illustrating the differential activity of MASLD genes associated with subsystems across various treatment groups. **(C)** Compass-score differential activity test between SET151 and the Control group, SET152 and the Control group, as well as SET162 and the Control group, respectively. RNA sequencing samples were prepared as quadruple.

To comprehensively characterize the metabolic alterations induced by JNK-IN-5A and its derivatives, we performed Compass metabolic activity analysis (Wagner et al., 2021) based on global transcriptomic profiling. The analysis was conducted using the RECON3D human metabolic network model, which encompasses 10,600 reactions, 5,835 metabolites and 2,248 genes. PCA of Compass output revealed three distinct metabolic clusters corresponding to the treatment groups: (1) SET-151, SET-152, and SET-162; (2) SET-130 and JNK-IN-5A; and (3) the untreated control group (Figure 4A). These results highlight the distinct metabolic states induced by the three most potent JNK-IN-5A derivatives compared to both the parental compound and the control.

We next performed differential reaction activity analysis to quantify metabolic alterations between the treatment and control groups. Cohen’s d effect sizes were calculated for each reaction, and significance was determined using Benjamini–Hochberg adjusted Wilcoxon rank-sum tests (*p* < 0.05), excluding reactions classified under the “Miscellaneous” subsystem. The SET-151, SET-152, and SET-162 groups exhibited 9,214, 9,580, and 9,945 significantly altered reactions, respectively. In contrast, no significantly changed reactions were detected in the SET-130 and JNK-IN-5A groups. As shown in Figure 4B, reactions related to lipid metabolism, including glycolysis, cholesterol metabolism, fatty acid synthesis, and pyruvate metabolism, were significantly altered in response to SET-151, SET-152, and SET-162 treatment, compared to the control group. Notably, SET-130 and JNK-IN-5A treatments did not induce significant changes in these key metabolic pathways (Supplementary Figure S1).

To further assess disease-relevant metabolic changes, we extracted 115 MASLD-associated reactions from the Compass results. The majority of these reactions were involved in cholesterol metabolism, fatty acid synthesis, and glycolysis/gluconeogenesis pathways (Figure 4C). Remarkably, activity levels of the three pyruvate kinase isoforms encoded by the PKLR gene were significantly reduced following SET-151, SET-152, and SET-162 treatment. This finding suggests that these JNK-IN-5A derivatives effectively disrupt pyruvate kinase-regulated glycolysis, contributing to their therapeutic effects in MASLD. Collectively, these metabolic analyses demonstrate that SET-151, SET-152, and SET-162 induce distinct and robust metabolic remodelling compared to JNK-IN-5A, with pronounced inhibition of lipids and pyruvate metabolism, supporting their superior potential as therapeutic candidates for MASLD. Among these hit compounds, SET-152 exhibited the strongest downregulation of glycolysis and cholesterol metabolism pathways. This suggests that SET-152 selected for the further *in vivo* stud.

### GLP (good laboratory Practice)-like toxicity assessment of SET-152 in rats demonstrates its safety

After testing the efficacy of SET-152 in an *in vitro* model of steatosis and revealing its MoA based on global transcriptomics and systems biology approach, we conducted a GLP-like 7-day oral tolerability study in Wistar rats to evaluate its safety at three dose levels (30, 100, and 300 mg/kg). The study included a vehicle control group (4 males and four females) and three dose groups, each consisting of three male and three female rats. The oral gavage dosing was administered once daily in the morning for seven consecutive days.

**FIGURE 5:**
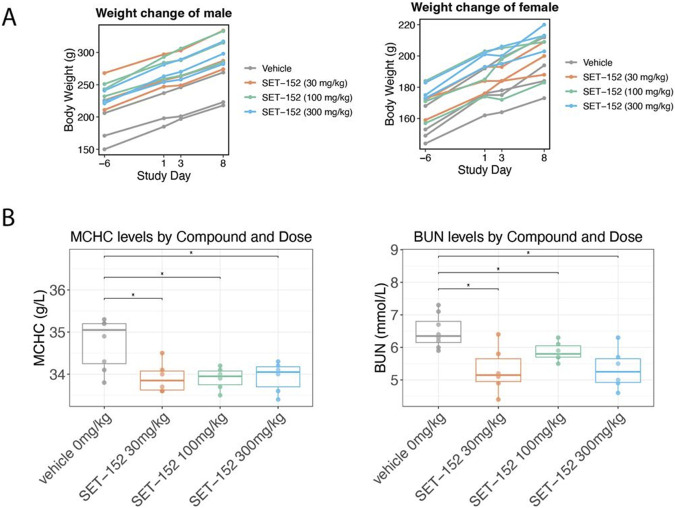
GLP-like toxicity study supporting the safety of the compound in rats. **(A)** Body weight changes over time across treatment groups. **(B)** Mean corpuscular haemoglobin concentration (MCHC) and blood urea nitrogen (BUN) levels showed statistically significant changes over time, but without a clear dose-dependent pattern. Vehicle (4 Male +4 Female), 30 mg/kg SET151 (3 Male +3 Female), 100 mg/kg SET151 (3 Male +3 Female), 300 mg/kg SET151 (3 Male +3 Female).

No clinical adverse effects were observed in any of the treated animals after 7 days. Body weight, haematology, and plasma chemistry analysis were evaluated. Treatment had no significant effect on body weight (Figure 5A). While mean red cell haemoglobin concentration (MCHC) and blood urea nitrogen (BUN) levels showed statistically significant changes in the treated animals, these changes were randomly distributed across dose groups and exhibited no dose-dependent effects (Figure 5B). These changes were considered incidental and unrelated to the treatment by the toxicology experts at RISE, Sweden. All other parameters remained within normal ranges, further supporting the safety of the SET-152 (Supplementary Figure S3).

### Evaluation of JNK-IN-5A and SET-152 in a high-fat, high-sucrose diet-induced *in vivo* MASH model

**FIGURE 6:**
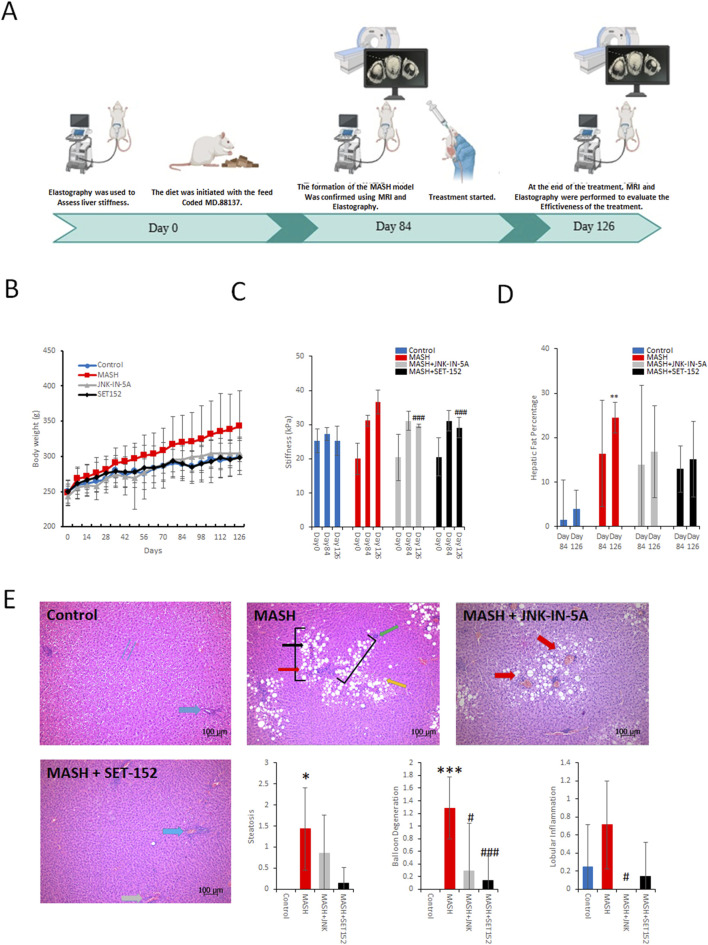
SET152 exhibited distinct therapeutic efficacy in reversing MASH *in vivo*. **(A)** Schematic overview of *in vivo* study. 6–8 weeks 29 SD rats (Control 8, MASH 7, MASH + JNK-IN-5A 7, and MASH + SET152 7) underwent MASH induction *via* a high-sucrose and fat diet for 84 days. JNK-5A-IN and SET152 were treated for the following 42 days. **(B)** Body weight was measured weekly over the 126 days experimental period. **(C)** Liver stiffness (kPa) was recorded at baseline (Day 0), after MASH induction (Day 84), and at the end of drug treatment (Day 126). **(D)** MRI measured liver fat content. **(E)** Haematoxylin and eosin (H&E) staining for liver tissue collected at day 126. Histological features are indicated as follows: portal area (blue arrow), hepatic cord (blue bracket), panlobular steatosis (black bracket), macrovesicular steatosis-macro droplet (black arrow), macrovesicular steatosis-micro droplet (red arrow), hepatocytes with ballooning degeneration (yellow arrow), lobular inflammation (green arrow), and central vein (gray arrow). Data are represented as mean±SD. Significance was tested using t-test. Group comparisons were conducted using one-way ANOVA followed by the *post hoc* Tukey test. * Represent statistical significance compared to the control group (*p < 0.05, **p < 0.01, ***p < 0.001). # Represent statistical significance relative to the MASH group (#*p* < 0.05, ##*p* < 0.01, ###*p* < 0.001).

To further assess the therapeutic potential of JNK-IN-5A and SET-152 in rats, we developed a high-fat, high-sucrose (HFHS) diet-induced MASH animal model (Figure 6A). Rats were fed the HFHS diet for 84 days (12 weeks) to induce MASH, which was confirmed using non-invasive methods, including two-dimensional (2D) shear wave elastography and magnetic resonance imaging (MRI).

Following MASH induction, rats were treated with either JNK-IN-5A or SET-152 at a dose level of 30 mg/kg *via* oral gavage for 42 days (6 weeks). Body weight was monitored weekly throughout the study (Figure 6B). By 126 days, the MASH group exhibited a significant increase in body weight (343 ± 50 g) compared to the control group (300 ± 25.6 g). Notably, the JNK-IN-5A (304 ± 23.9 g) and SET-152 (298 ± 24.3 g) treatment groups maintained their body weights comparable to those of healthy controls.

Liver stiffness, a marker of fibrosis, was evaluated using 2D shear wave elastography on day 126 (Figure 6C). The MASH group exhibited significantly increased liver stiffness (36.7 ± 3.5 kPa) compared to controls (25.2 ± 4.3 kPa). Treatment with JNK-IN-5A (29.2 ± 0.4 kPa) and SET-152 (29.1 ± 2.9 kPa) significantly reduced liver stiffness compared to the untreated MASH group. Hepatic fat content was assessed by MRI at day 84 (post-MASH induction) and day 126 (following treatment) (Figure 6D). 42 days of treatment with JNK-IN-5A (16.8% ± 10.3%) and SET-152 (15.0% ± 8.5%) significantly reduced hepatic fat accumulation compared to the untreated MASH group (24.5% ± 3.4%). Histopathological analyses were performed on liver tissues using haematoxylin and eosin (H&E) staining (Figure 6E). Steatosis, ballooning degeneration, and lobular inflammation were scored according to standard criteria: steatosis (0–3), ballooning (0–2), and inflammation (0–3). The MASH group displayed significant pathological features, with elevated steatosis (1.43 ± 0.98), ballooning degeneration (1.29 ± 0.49), and lobular inflammation (0.71 ± 0.49) scores. JNK-IN-5A treatment reduced steatosis (0.86 ± 0.90), ballooning degeneration (0.29 ± 0.76), and eliminated lobular inflammation. Remarkably, SET-152 treatment led to a 90% reduction in steatosis (0.14 ± 0.38), an 89% reduction in ballooning degeneration (0.14 ± 0.38), and an 80% reduction in lobular inflammation (0.14 ± 0.38).

Blood biochemistry analyses were conducted at the end of the 126 days study to evaluate liver function and metabolic health (Table 1). The MASH group exhibited significant elevations in serum alanine aminotransferase (ALT) and TAG levels, indicative of liver injury and dyslipidaemia. Treatment with both JNK-IN-5A and SET-152 significantly reduced ALT and TAG levels, indicating an improvement in liver damage and metabolic dysfunction. To evaluate the safety of the compound in the MASH model, we performed a micronucleus (MN) assay to assess genotoxicity and conducted haematological analyses (Table S1). No significant differences in MN frequency were observed among the control, MASH, MASH + JNK-IN-5A, and MASH + SET-152 groups, indicating no detectable genotoxicity. Similarly, haematological parameters remained within normal ranges across all groups, suggesting that neither compound induced haematological toxicity (Table 2). Collectively, these findings demonstrate that both JNK-IN-5A and its derivative SET-152 effectively attenuate MASH-related pathological features, including hepatic fat accumulation, liver stiffness, inflammation, and biochemical markers, without inducing observable toxicity in an *in vivo* MASH model. Notably, SET-152 exhibited promising therapeutic efficacy, supporting its potential candidate for MASH treatment.

**TABLE 1:** Blood biochemistry of liver injury and MASLD progression.

Groups	Control	MASH	MASH + JNK-IN-5A	MASH + SET152
ALP (U/L)	158.71 ± 26.71	104.29 ± 27,35^**^	93.29 ± 27,67^***^	118.57 ± 21.97
AST (U/L)	236.11 ± 24.65	262.86 ± 41.5	225.39 ± 90.87	216.6 ± 40.84
ALT (U/L)	52.01 ± 17.5	118.63 ± 32,39^***^	32.68 ± 6,31^###^	38.37 ± 15,65^###^
LDH (U/L)	1951.71 ± 485.58	2523.29 ± 515.64	1623.67 ± 541.00	2212.83 ± 389.82
Triglyceride (mg/dL)	81.71 ± 18.22	184.43 ± 53,89^***^	108.43 ± 31,66^##^	96.14 ± 37,73^###^
Total cholesterol (mg/dL)	61.57 ± 9.64	76.86 ± 4.91	78.86 ± 19.80	54.86 ± 8,30^#^
HDL cholesterol (mg/dL)	41.00 ± 6.51	38.86 ± 6.31	42.14 ± 13.12	36.71 ± 8.18
LDL cholesterol (mg/dL)	10.57 ± 2.51	12.86 ± 3.18	11.29 ± 2.21	8.43 ± 2.44
Total protein (g/dL)	7.21 ± 0.50	7.56 ± 0.38	8.16 ± 0,99^*^	7.54 ± 0.54
Albumin (g/dL)	3.20 ± 0.21	3.63 ± 0.22	3.81 ± 0,45^*^	3.49 ± 0.30
Glucose (mg/dL)	145.57 ± 31.65	205.00 ± 88.24	208.43 ± 64.02	228.57 ± 165.46
Total bilirubin (mg/dL)	0.09 ± 0.05	0.11 ± 0.07	0.10 ± 0.08	0.10 ± 0.05
Creatine (mg/dL)	0.44 ± 0.03	0.53 ± 0.14	0.53 ± 0.12	0.43 ± 0.16
Uric acid (mg/dL)	0.98 ± 0.35	1.33 ± 0.24	1.61 ± 0.93	1.35 ± 0.45

Serum biochemical markers were measured to evaluate liver function and metabolic alterations associated with MASH induction and treatment.

Data are represented as mean±SD. Significance was tested using t-test. Group comparisons were conducted using one-way ANOVA followed by the *post hoc* Tukey test. *p value < 0.05, #*p* value < 0.05 to MASH group.

**TABLE 2:** Blood haematology following MASH induction and treatment.

Groups	Control	MASH	MASH + JNK-IN-5A	MASH + SET152
PCT (%)	0.68 ± 0.12	0.71 ± 0.10	0.77 ± 0.14	0.72 ± 0.16
PLT (10^3/μL)	845.57 ± 158.62	871.29 ± 128.53	947.71 ± 123.89	904.86 ± 140.16
MCHC (g/dL)	28.24 ± 0.96	28.97 ± 0.69	29.27 ± 1.14	29.71 ± 1.11
MCH (fL)	17.41 ± 0.60	17.84 ± 0.59	18.04 ± 0.48	17.81 ± 0.60
MCV (fL)	61.67 ± 2.07	61.51 ± 1.66	61.69 ± 2.87	60.11 ± 2.25
HCT (%)	54.40 ± 2.67	55.51 ± 2.31	55.41 ± 5.42	51.99 ± 3.93
HGB (g/dL)	15.19 ± 1.27	16.09 ± 0.76	16.20 ± 1.51	15.40 ± 1.27
RBC (10^6/μL)	8.83 ± 0.60	9.04 ± 0.52	8.98 ± 0.65	8.66 ± 0.73
WBC (10^3/μL)	5.81 ± 2.27	4.70 ± 1.50	4.27 ± 1.67	5.75 ± 3.07

Haematological analysis was performed to evaluate systemic effects of MASH, and subsequent treatment with JNK-IN-5A, or SET152. Parameters include platelet count (PLT), procalcitonin (PCT), mean corpuscular haemoglobin concentration (MCHC), mean corpuscular haemoglobin (MCH), mean corpuscular volume (MCV), haematocrit (HCT), haemoglobin (HGB), red blood cell count (RBC), and white blood cell count (WBC).

Data are represented as mean±SD., Group comparisons were conducted using one-way ANOVA, followed by the *post hoc* Tukey test.

## Discussion

The dysregulation of hepatic lipid metabolism, particularly increased DNL, is a central feature of MASLD and its progressive form, MASH. Multiple enzymes in the DNL pathway have been identified as therapeutic targets, with several candidates showing promising results in clinical and preclinical studies. Denifanstat (TVB-2640), a FASN inhibitor currently in a Phase 2 b clinical trial, suppresses FASN activity and reduces hepatic TAG accumulation by inhibiting DNL. In addition to its anti-steatotic effects, Denifanstat has been shown to attenuate steatohepatitis through the deactivation of hepatic stellate cells (Loomba et al., 2021a; Syed-Abdul et al., 2020; O’Farrell et al., 2022) and has demonstrated anti-cancer activity in models of lung carcinoma, breast cancer, astrocytoma, and colon cancer (Fries et al., 2023; Kelly et al., 2023; Falchook et al., 2021; Cao et al., 2022). Similarly, Firsocostat, an ACACA inhibitor that has completed a Phase 2 clinical trial, effectively reduces hepatic DNL (Kirby et al., 2021) and improves liver fibrosis (Loomba et al., 2021b; Alkhouri et al., 2022; Alkhouri et al., 2020). Several preclinical ACACA inhibitors, including CP-640186 (Corominas-Faja et al., 2014; Hess et al., 2010), Soraphen A (Corominas-Faja et al., 2014; Schreurs et al., 2009), and TOFA (Li et al., 2013), have also been reported to reduce weight gain, hepatic steatosis, and inflammation, while exhibiting anti-cancer properties. The anti-cancer effects of the FASN and ACACA-targeting molecules are attributed mainly to the suppression of lipid synthesis, a metabolic pathway essential for both energy storage and the production of key cellular components, including membranes and signalling molecules. Importantly, lipid biosynthesis is often markedly upregulated in cancer cells to support rapid proliferation, a phenomenon associated with the Warburg effect (Burns and Manda, 2017).

Our study expands on this therapeutic paradigm by identifying PKL isoform, encoded by the PKLR gene, as a novel, highly disease-associated target for MASLD and MASH. Systems biology analysis, combined with global transcriptomic profiling, has identified PKLR as one of the most significantly associated genes with MASLD and HCC (Lee et al., 2017). PKLR catalyses the final step of glycolysis, linking glucose and pyruvate metabolism to the DNL pathway by providing precursors for fatty acid synthesis. Notably, the inhibition of PKLR is expected to suppress both glycolysis-derived substrate availability and downstream lipogenesis, offering a multifaceted approach to target metabolic dysregulation in MASLD.

In this context, we applied a computational drug repurposing approach and identified JNK-IN-5A as a small molecule that modulates PKLR expression. Building on this, we synthesized and evaluated a series of JNK-IN-5A derivatives (SET-151, SET-152, SET-162, and SET-130), three of which (SET-151, SET-152, and SET-162) exhibited superior anti-steatotic efficacy. Our data demonstrate that these derivatives not only downregulate PKLR expression but also suppress the expression of critical enzymes in the DNL pathway (FASN, ACACA) and SCD. SCD is a well-established therapeutic target for MASLD and certain cancers (Sun et al., 2024; Jeyakumar and Vajreswari, 2022; Min and Kim, 2023; Sen et al., 2023; Katoh et al., 2022). Pharmacological inhibition of SCD has been shown to exert beneficial effects in metabolic diseases and cancer. For example, the SCD inhibitor E6446 suppresses both SCD expression and the transcription factor ATF3, leading to impaired adipogenic differentiation and reduced hepatic lipogenesis (Wang et al., 2023). Furthermore, Ascenzi et al. demonstrated the pivotal role of SCD as a key therapeutic target in cancer (Ascenzi et al., 2021). Inhibition of SCD reduces MUFA synthesis, resulting in the accumulation of SFAs. Elevated intracellular SFA levels are known to trigger cellular stress responses, including enhanced autophagy. Excessive autophagy can, in turn, promote apoptosis, lipotoxicity, and ferroptosis. Based on these established mechanisms, we hypothesize that the marked reduction of SCD protein expression observed following treatment with JNK-IN-5A and its derivatives may contribute to the reduced cell viability seen in the HepG2 DNL steatosis model. The simultaneous downregulation of PKLR, FASN, ACACA, and SCD by our compounds offers a unique multiple-targeting strategy that disrupts both substrate supply and enzymatic execution of lipid biosynthesis.

Further transcriptomic and metabolic modelling revealed that SET-151, SET-152, and SET-162 induced broad transcriptional changes beyond PKLR suppression, downregulating pathways essential for MASLD and MASH progression, including pyruvate metabolism, bile acid biosynthesis, fatty acid metabolism, and glycolysis. These transcriptomic effects were also revealed in Compass metabolic activity analysis, which demonstrated that only these three derivatives, but not JNK-IN-5A or SET-130, significantly altered reaction activities within key lipid metabolic pathways, including glycolysis and fatty acid synthesis, which are crucial biological pathways in MASLD and MASH progression (Heeren and Scheja, 2021). Of particular interest, activity levels of all three pyruvate kinase isoforms were reduced, suggesting that the observed metabolic reprogramming is directly linked to PKLR downregulation and impaired glycolytic flux fuelling DNL.

Mechanistically, the link between JNK signalling, SREBP-1C activation, and DNL regulation provides a plausible explanation for the broad inhibitory effects of JNK-IN-5A and its derivatives. Previous studies have shown that MAPK-mediated phosphorylation of SREBP-1C is critical for its activation and nuclear translocation, enabling the transcription of lipogenic genes such as FASN and ACACA (Kotzka et al., 2011). Preventing phosphorylation by JNKs has been shown to protect against hepatic steatosis and visceral obesity in mice (Ko et al., 2012). However, further studies are needed to elucidate the precise regulatory mechanisms underlying the DNL pathway inhibition mediated by JNK-IN-5A and its derivatives. Our data support this model, showing that SET-151, SET-152, and SET-162 more potently reduce SREBP-1C and ChREBP levels than JNK-IN-5A, accompanied by a more substantial reduction in downstream DNL enzymes.

Importantly, *in vivo* studies using a HFHS diet-induced MASH rat model demonstrated that JNK-IN-5A and SET-152 reduced hepatic fat accumulation, liver stiffness, and key histological features of MASH, including steatosis, ballooning degeneration, and lobular inflammation. SET-152, in particular, showed more profound anti-steatotic effect *via* inhibition of DNL pathway than the reference compound, consistent with its *in vitro* activity. These beneficial effects were achieved without evidence of genotoxicity or haematological toxicity, underscoring the potential therapeutic agent. Further mechanistic studies are warranted to elucidate the interplay between JNK signalling, PKLR expression, and SREBP-1C regulation, and to explore the translational potential of these compounds in human clinical settings.

Collectively, our findings identify PKLR as a promising, previously underexplored therapeutic target for MASLD and MASH. The development of SET-152 is capable of simultaneously suppressing PKLR, FASN, ACACA, and SCD, representing a novel, multi-targeted approach to disrupt the metabolic underpinnings of hepatic steatosis. The promising efficacy of SET-152, as demonstrated through *in vitro* and *in vivo* analyses, highlights its potential as a first-in-class DNL and lipid metabolism modulating compound for MASLD and MASH therapy.

## Limitations of the study

Our study extensively showed that JNK-IN-5A and its derivatives, specifically SET151, SET152, and SET162 attenuate hepatic steatosis *via* inhibition of DNL pathway *in vitro* and *in vivo*. Despite this vast range of anti-steatosis effect, there are still works to prove to develop new drug. Firstly, much more strong mechanism study which clearly showing interaction between JNK-IN-5A derivatives and PKLR. We indirectly showed compounds interact with JNK2 *via* CETSA. Further analysis such as molecular docking, pull-down assay, and Co-IP will give strong drug MoA. We compared our compounds with resmetirom at *in vitro* HepG2 DNL steatosis model in this research. HepG2 DNL steatosis model showed well established lipogenic fatty acid accumulation, but it is not sufficient to show therapeutic effect of resmetirom. Resmetirom is a THR-β membrane receptor agonist which correlated thyroid hormone and has systemic metabolic effects *via* modulating lipid oxidation and bile acid synthesis. Test resmetirom and show therapeutic effect at HepG2 DNL *in vitro* model is meaningful but still there is limitations. GLP-like toxicity study showed non-significant toxicity *in vivo*, however, it still remains at preliminary tolerability assessment. We need more longer period toxicological study and also pharmacokinetics evaluation. SET-152 showed anti-steatotic effect and attenuation of liver stiffness on rat MASLD model. To move next drug discovery step, there are still more *in vivo* validation. *In vivo* transcriptomics for steatosis and liver fibrosis, and histological analysis such as Sirius Red or Masson’s trichrome staining, with various dose dependent manner.

## Data Availability

The original contributions presented in the study are included in the article/Supplementary Material, further inquiries can be directed to the corresponding author.
