# Supplementary material for: Targeting PKLR and lipogenic enzymes through JNK inhibition to develop a therapeutic strategy for MASLD and MASH
Source: Front Pharmacol. 2026 Jul 1;17:1823203. doi: 10.3389/fphar.2026.1823203 (PMC13369619; doi:10.3389/fphar.2026.1823203)
Supplement: Supplementary file 1 [file DataSheet1.pdf]

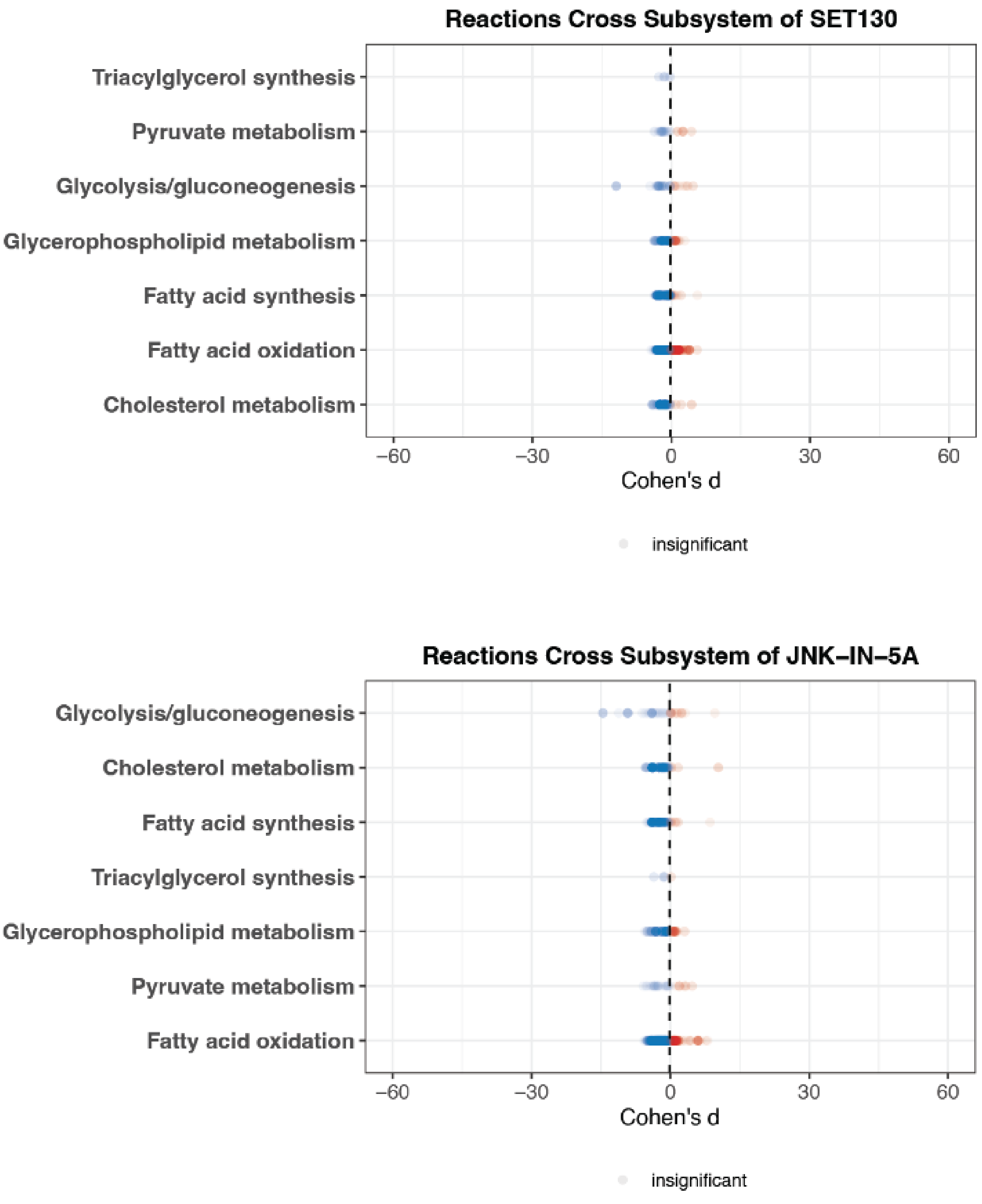

Figure S1

Compass-score differential activity test between SET130 and the Control group, JNK-IN-5A and the Control group, respectively.

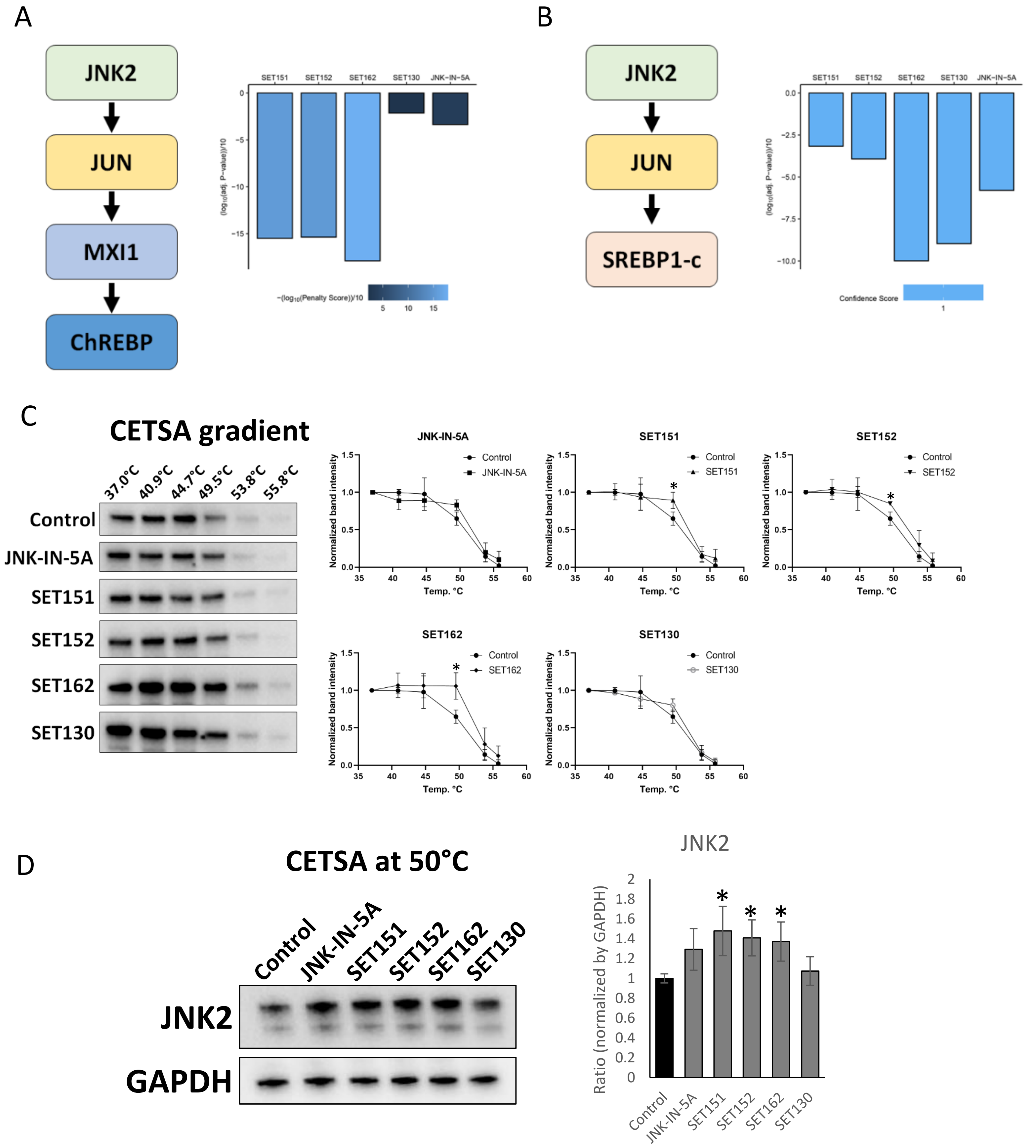

Figure S2

(A) Predicted mechanism of action for drugs regulating chREBP. Penalty scores and gene expression significance are shown by the bar plot.

(B) Predicted mechanism of action for drugs regulating SREBP-C. Confidence scores and gene expression significance are shown by the bar plot.

(C) Cellular thermal shift assay (CETSA). HepG2 cell lysate were treated at 20μM compounds and had heat shock with gradient temperature for 5min.

(D) Cellular thermal shift assay (CETSA). Cells were treated at 20μM for 2hr and had heat shock at 50°C for 3min.

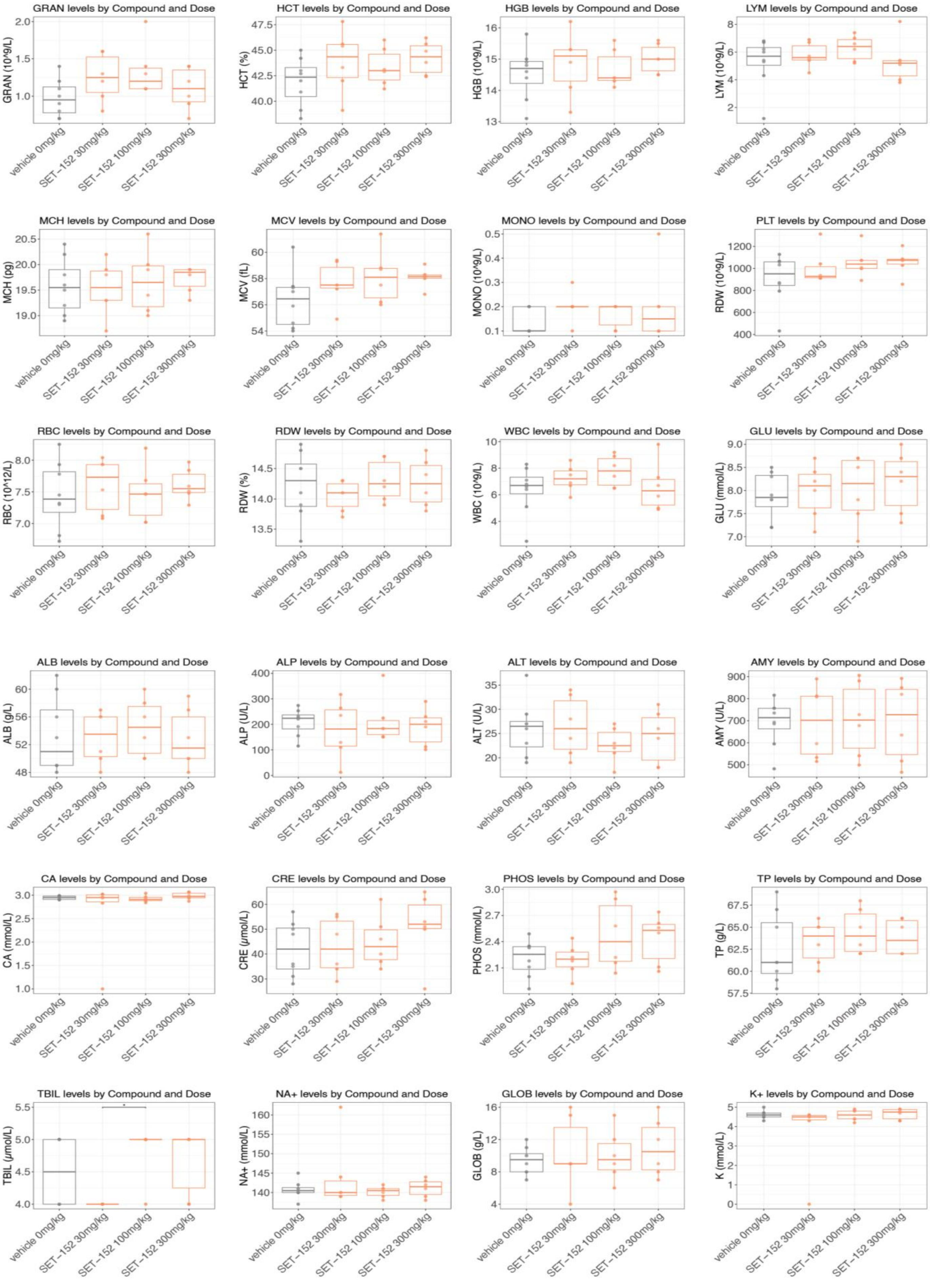

Figure S3

Hematology and plasma chemistry parameters remained within normal ranges, supporting the overall safety profile of the compound.

Table S1. Micronucleus assay.

Groups	MN frequency (%)
**Control**	3,68±0,84
**MASH**	3,42±1,22
**MASH+JNK-5A**	3,18±0,37
**MASH+SET152**	2,76±0,15

Femur bones marrow micronucleus ratio was calculated.
Data are represented as mean +/- SD.

Figure 1D

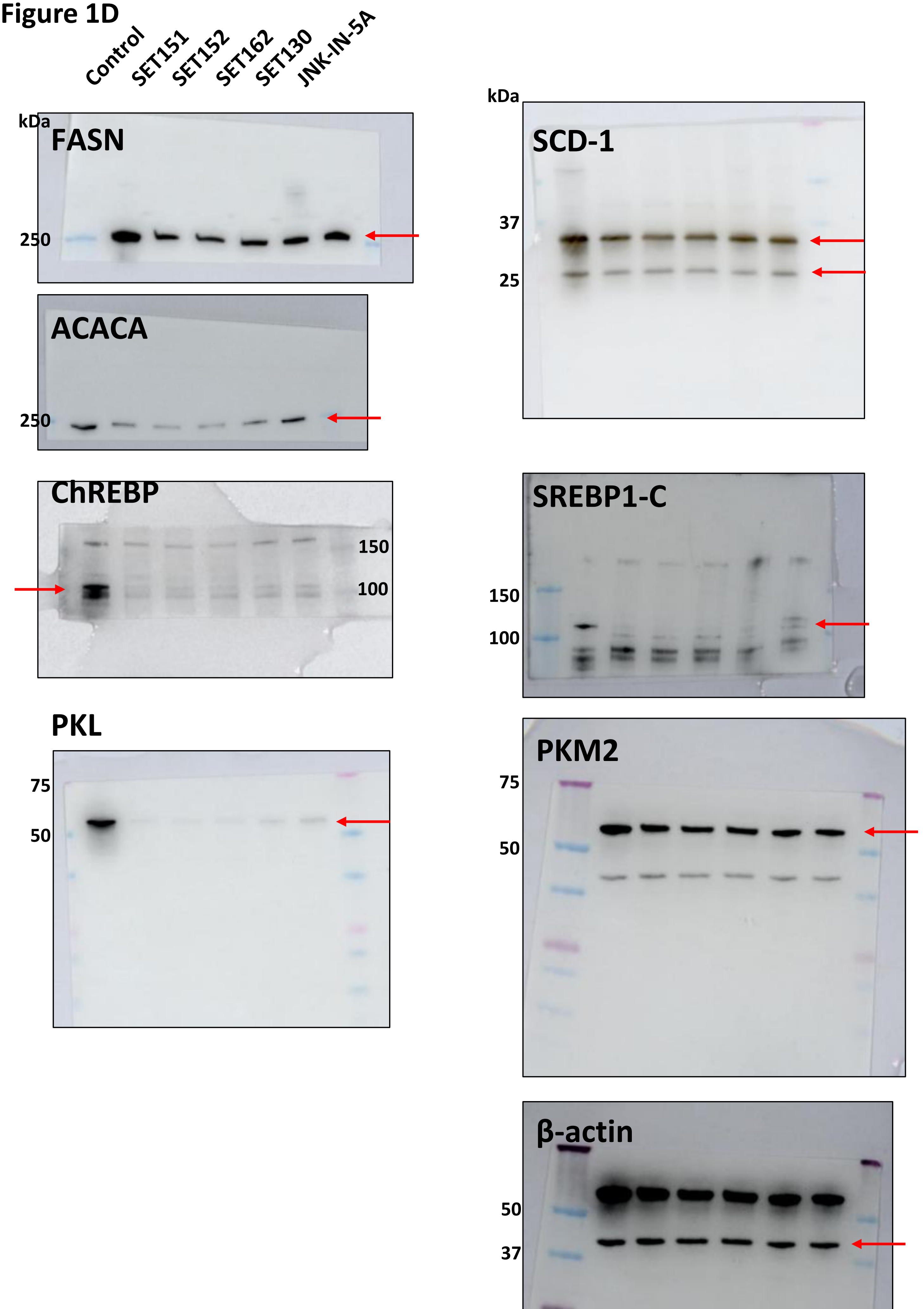
Control
SET151
SET152
SET162
SET130
JNK-IN-5A
kDa
FASN
250
ACACA
250
ChREBP
150
100
PKL
75
50
kDa
SCD-1
37
25
SREBP1-C
150
100
PKM2
75
50
β-actin
50
37

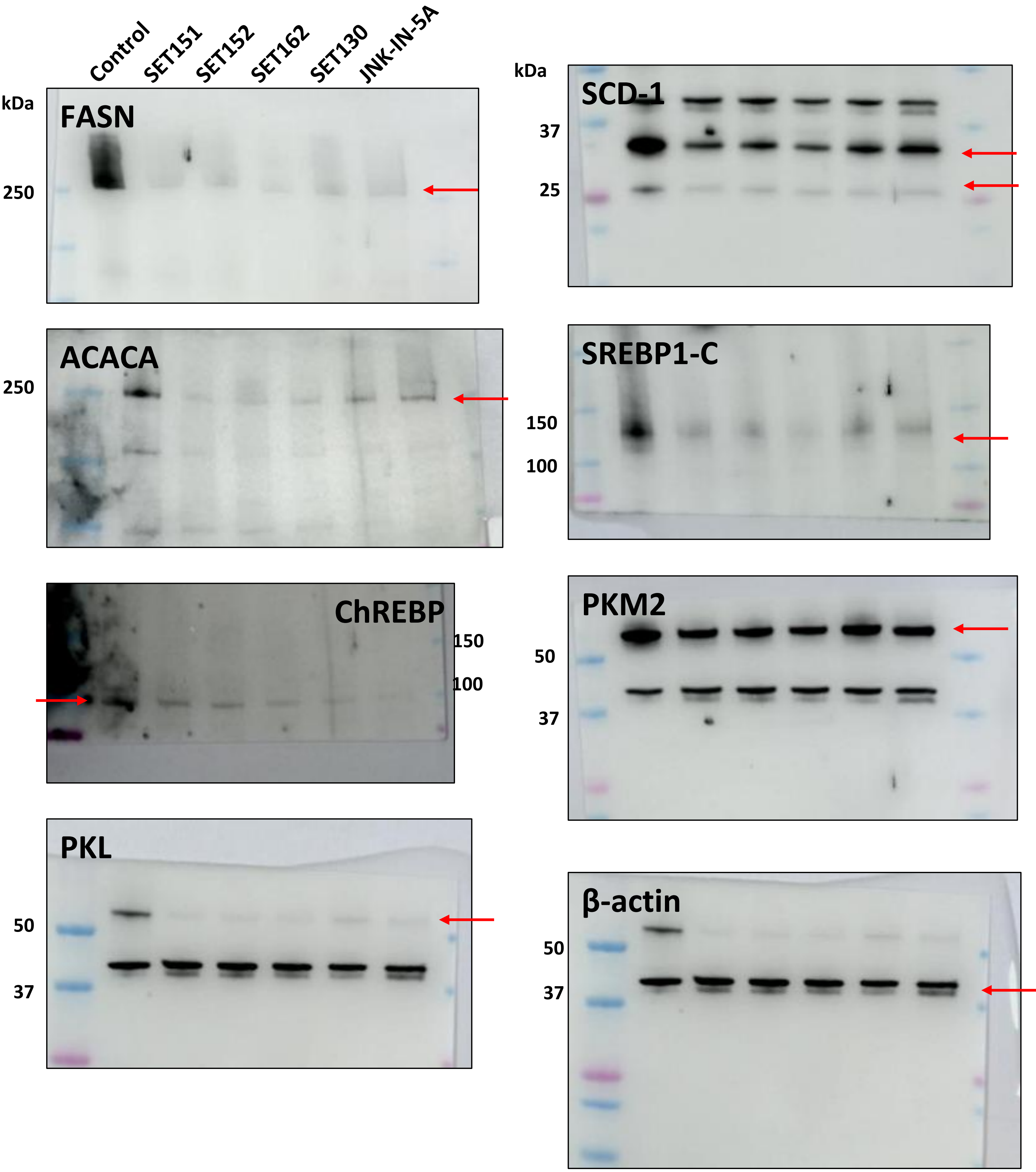

Control
SET151
SET152
SET162
SET130
JNK-IN-5A
kDa
FASN
250
ACACA
250
ChREBP
150
100
PKL
50
37
kDa
SCD-1
37
25
SREBP1-C
150
100
PKM2
50
37
β-actin
50
37

Figure 1D

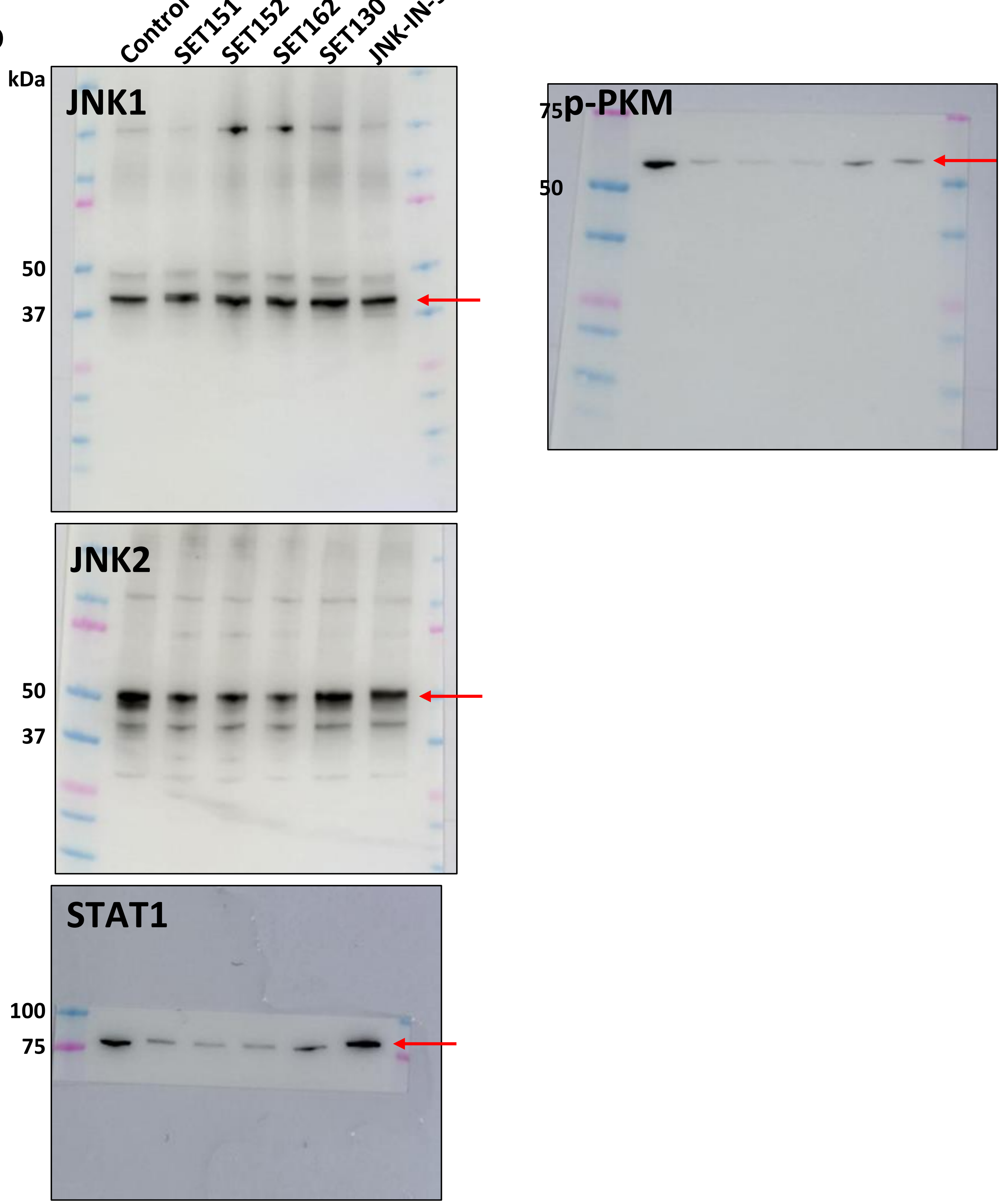

Control
SET151
SET152
SET162
SET130
JNK-IN-
kDa
JNK1
50
37
JNK2
50
37
STAT1
100
75
p-PKM
75
50

Figure 2B

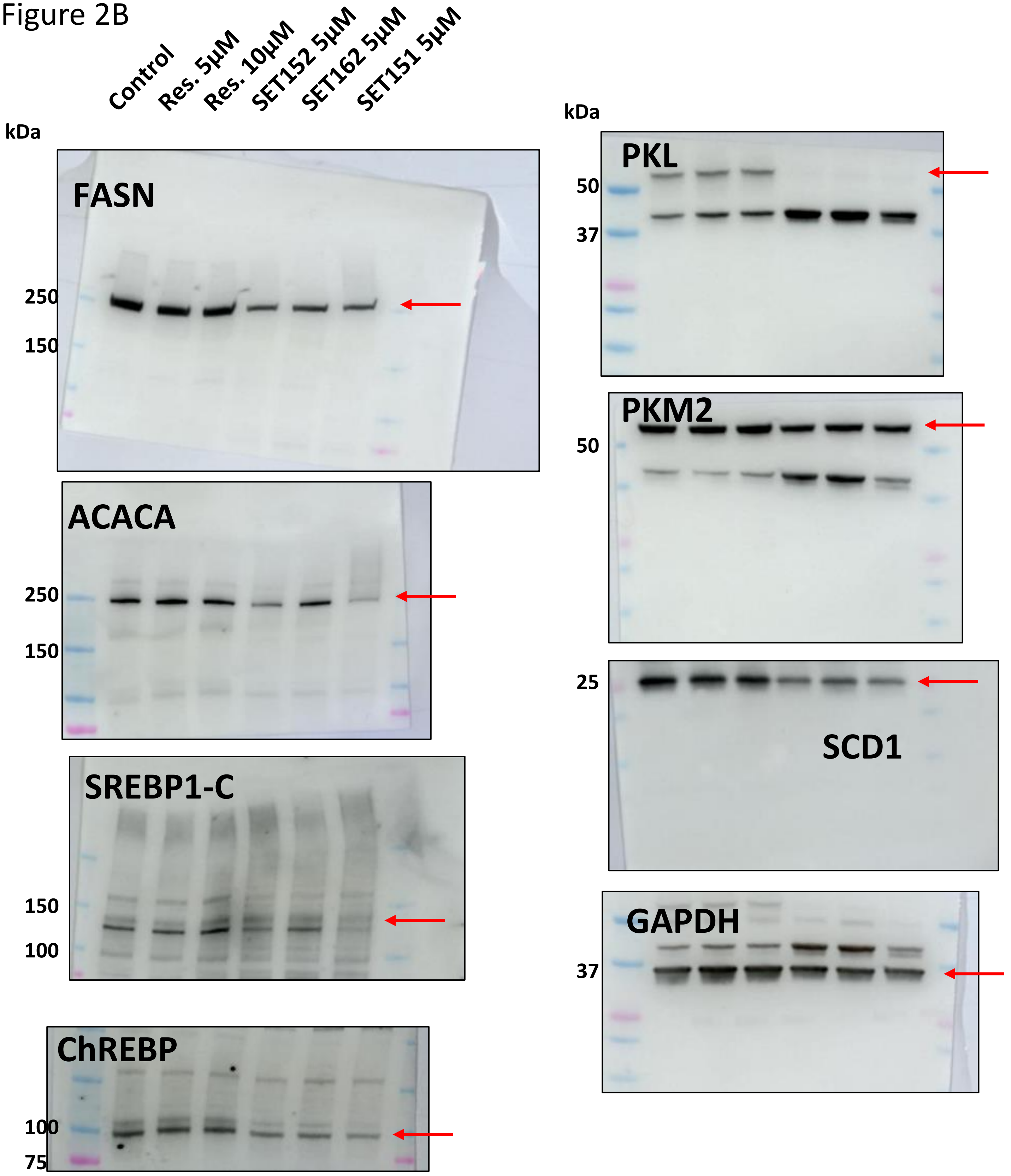
Control
Res. 5µM
Res. 10µM
SET152 5µM
SET162 5µM
SET151 5µM
kDa
FASN
250
150
ACACA
250
150
SREBP1-C
150
100
ChREBP
100
75
kDa
PKL
50
37
PKM2
50
25
SCD1
GAPDH
37

Figure S2C

JNK2

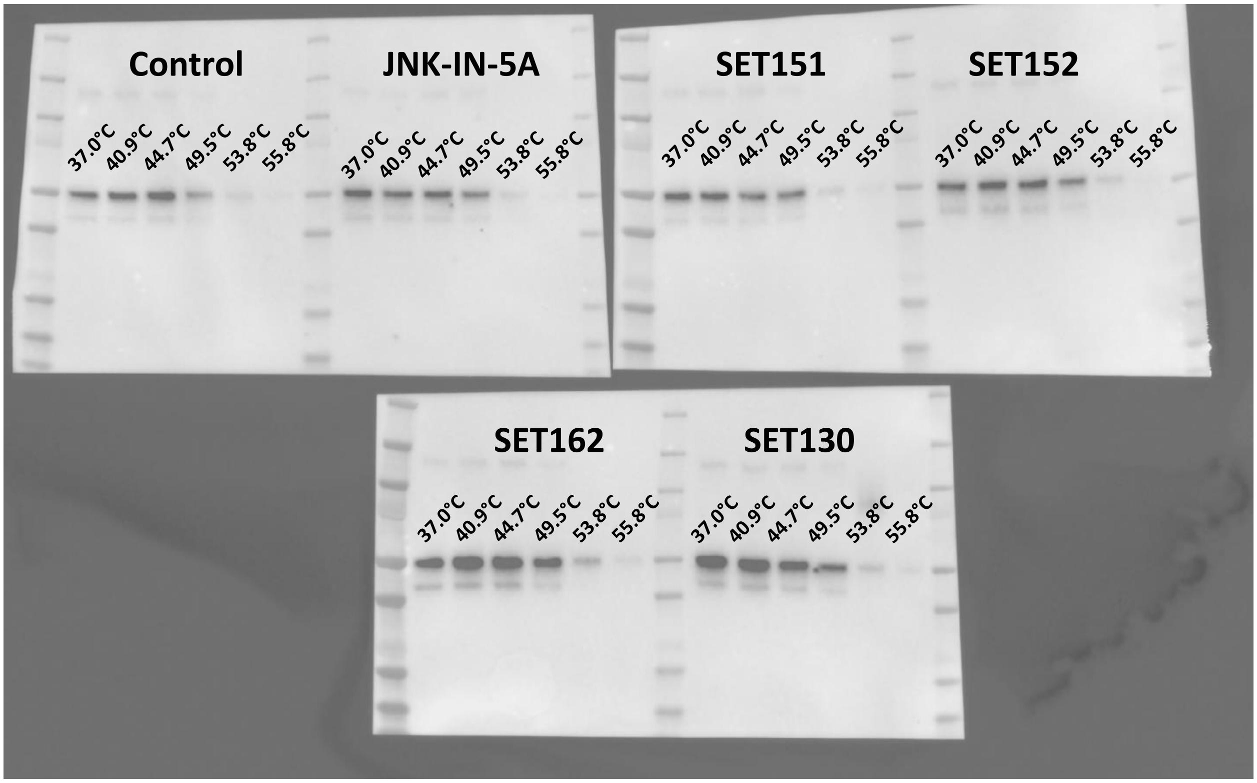

GAPDH

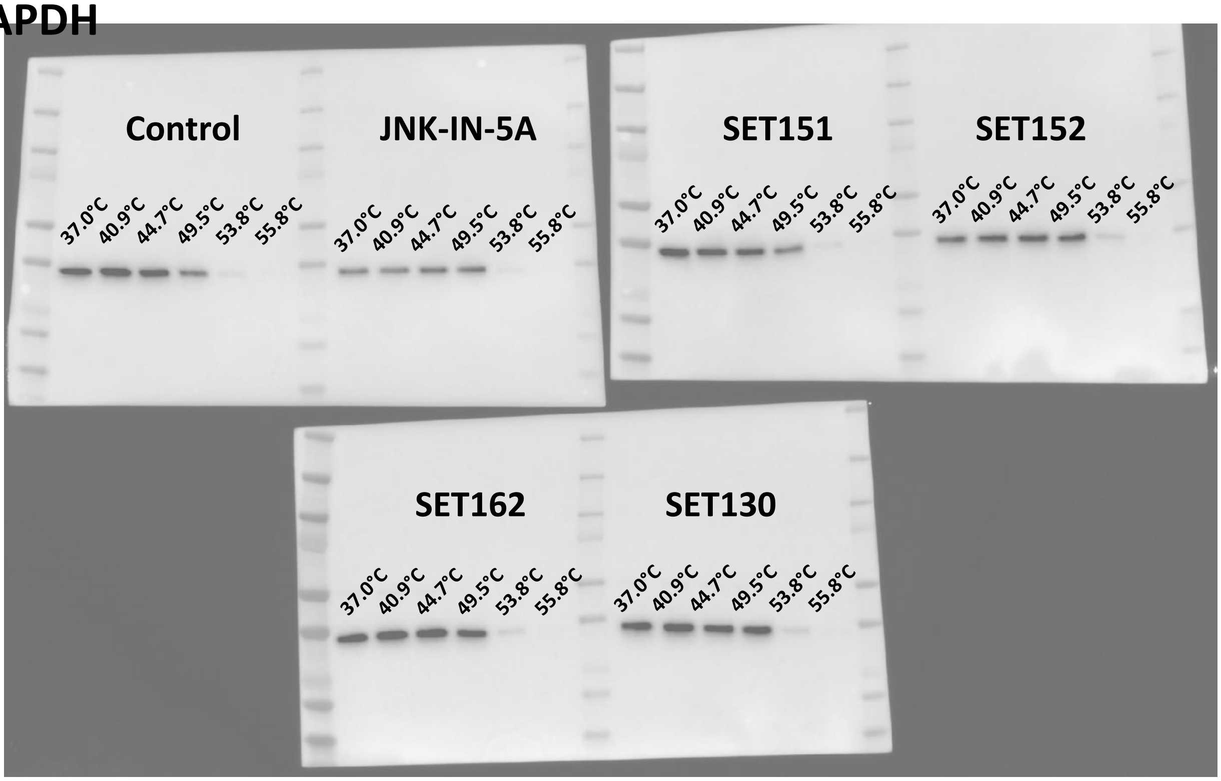

Figure S2C

JNK2

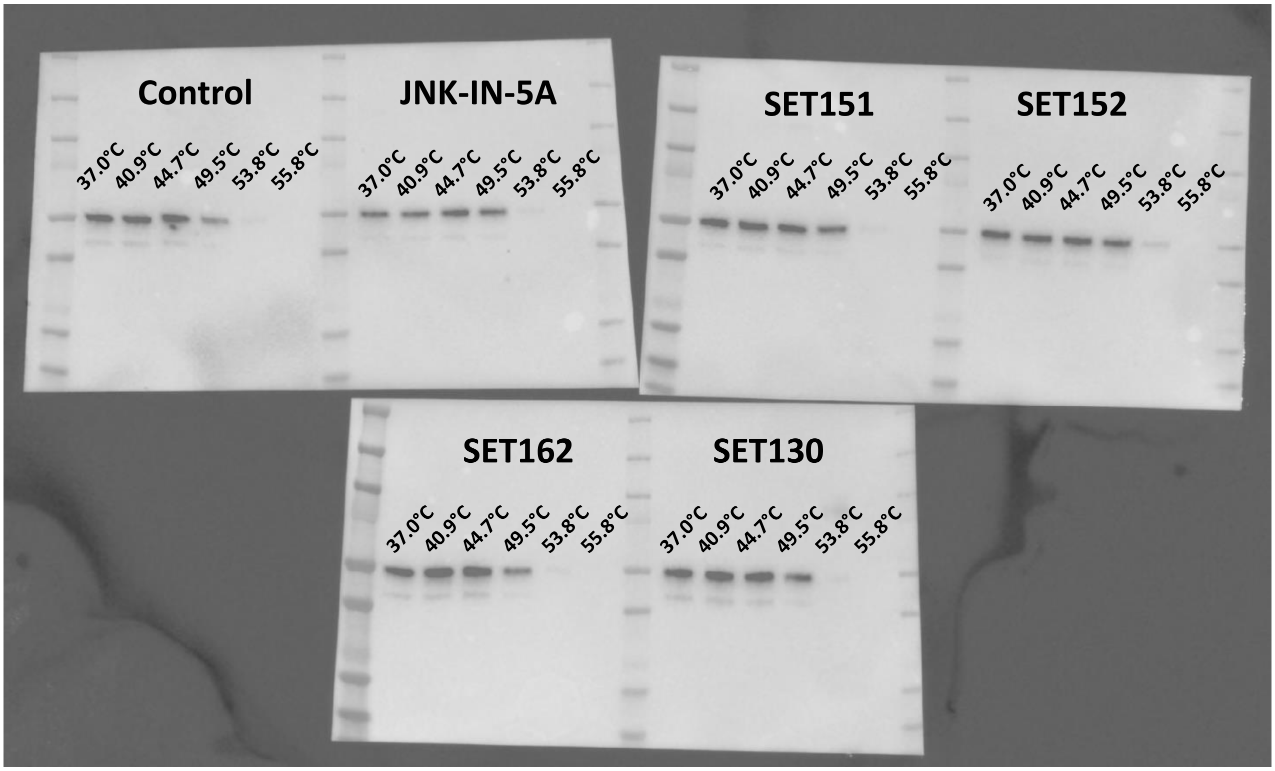

GAPDH

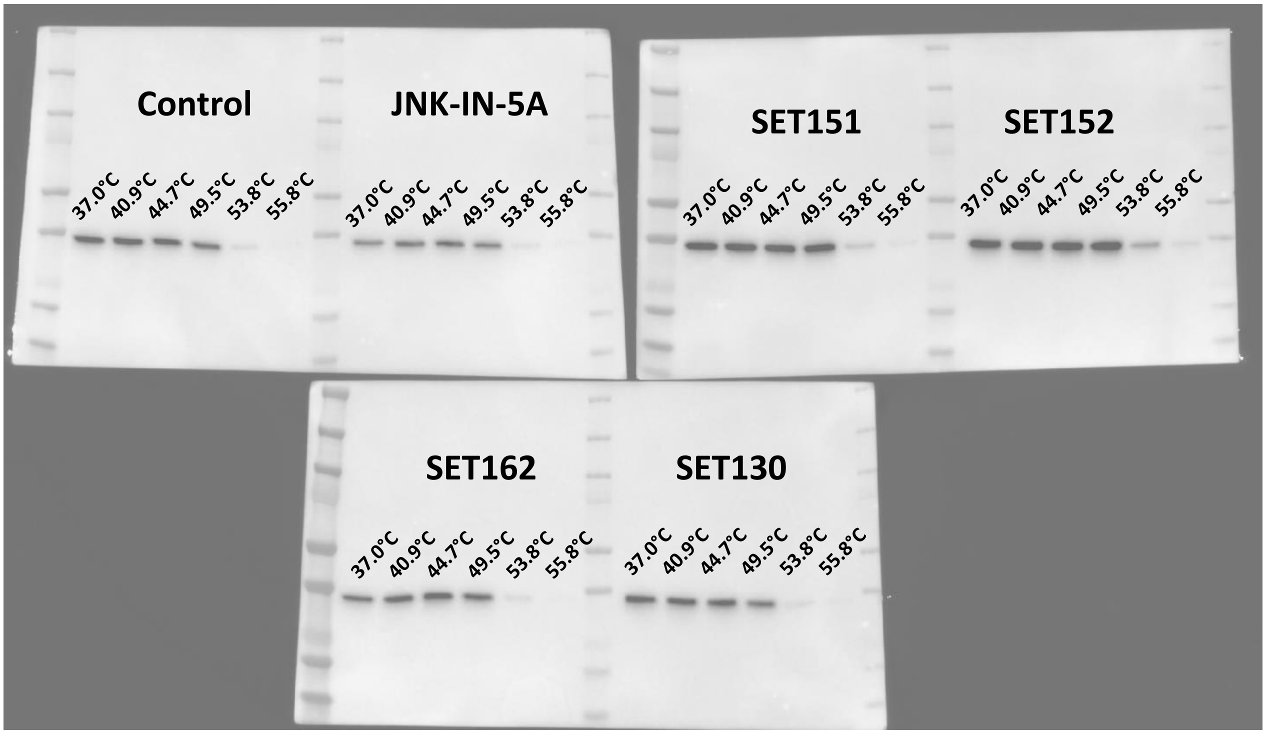

Figure S2C

JNK2

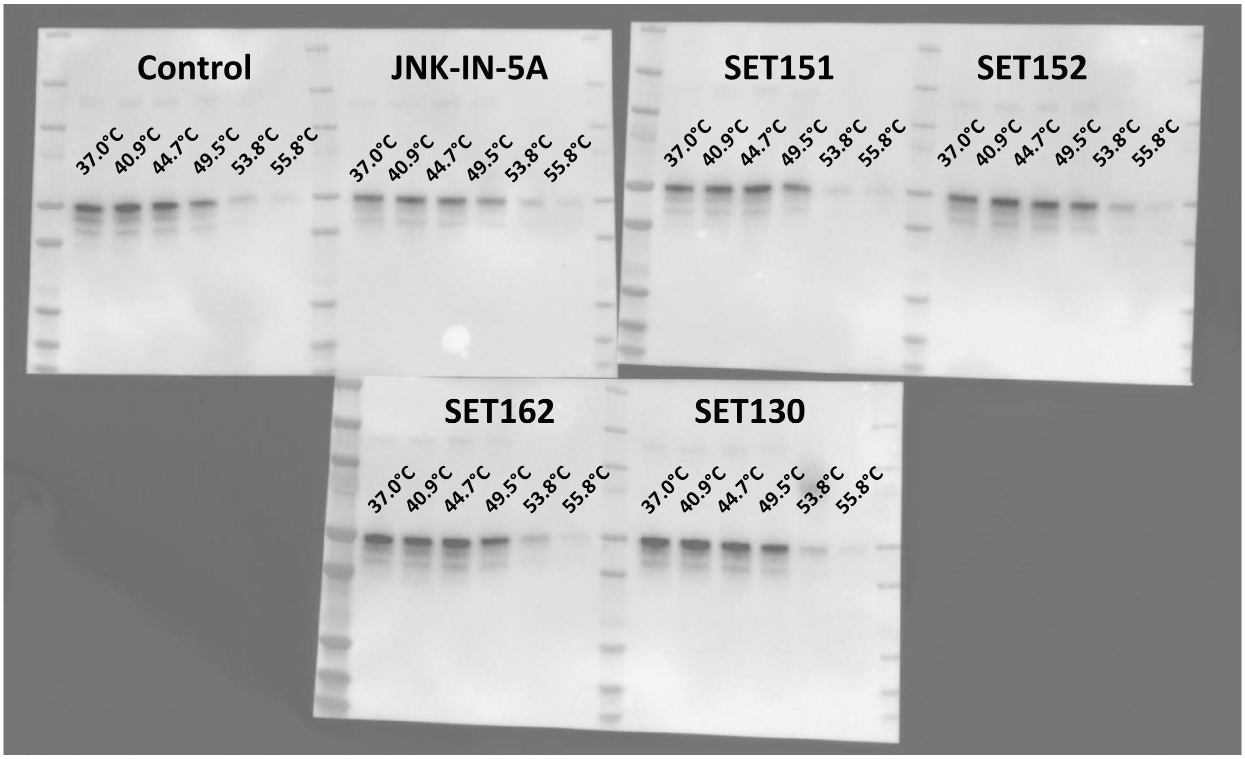

GAPDH

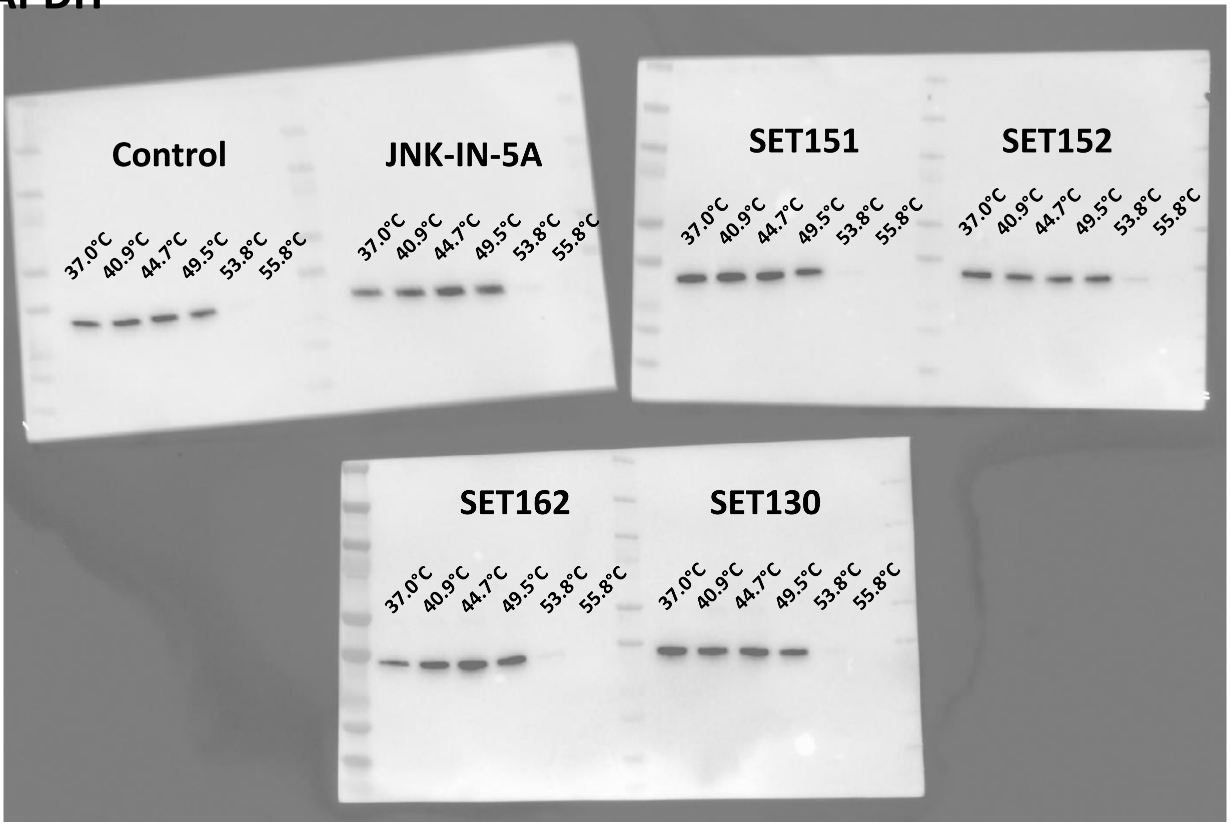

Figure S2D

JNK2

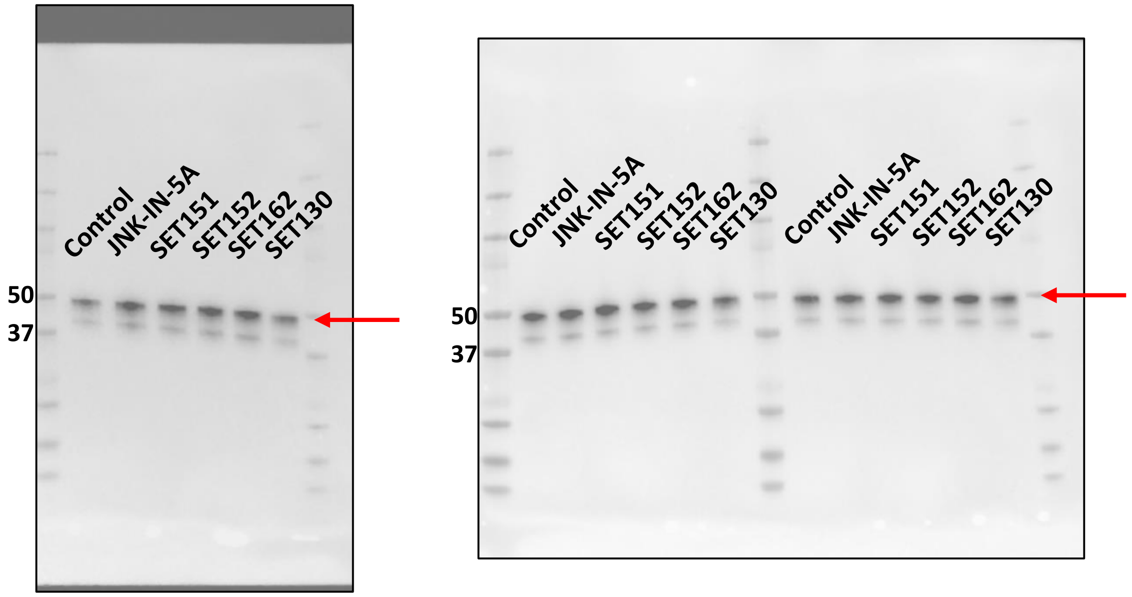

GAPDH

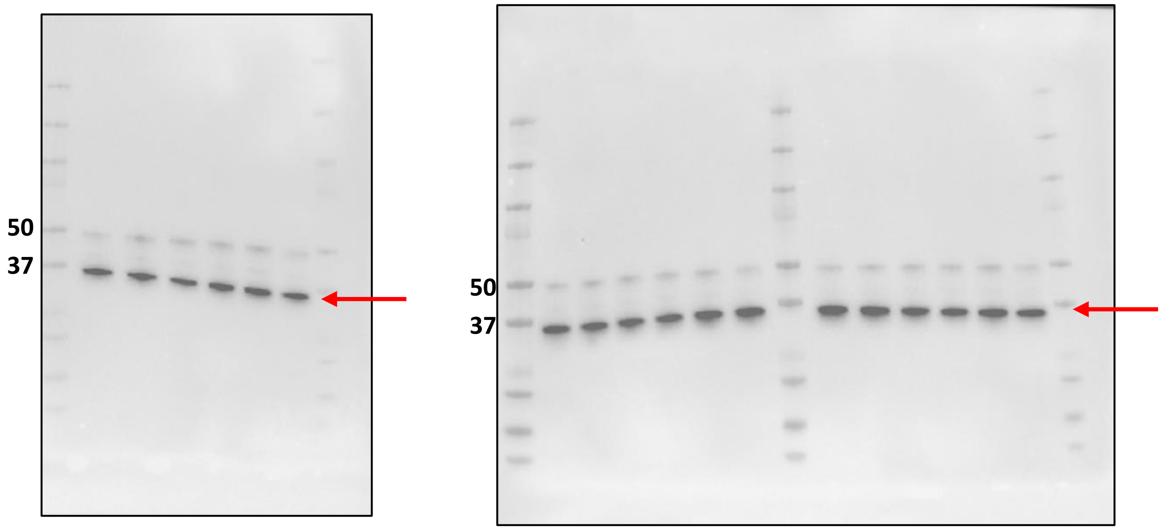

Figure 1E

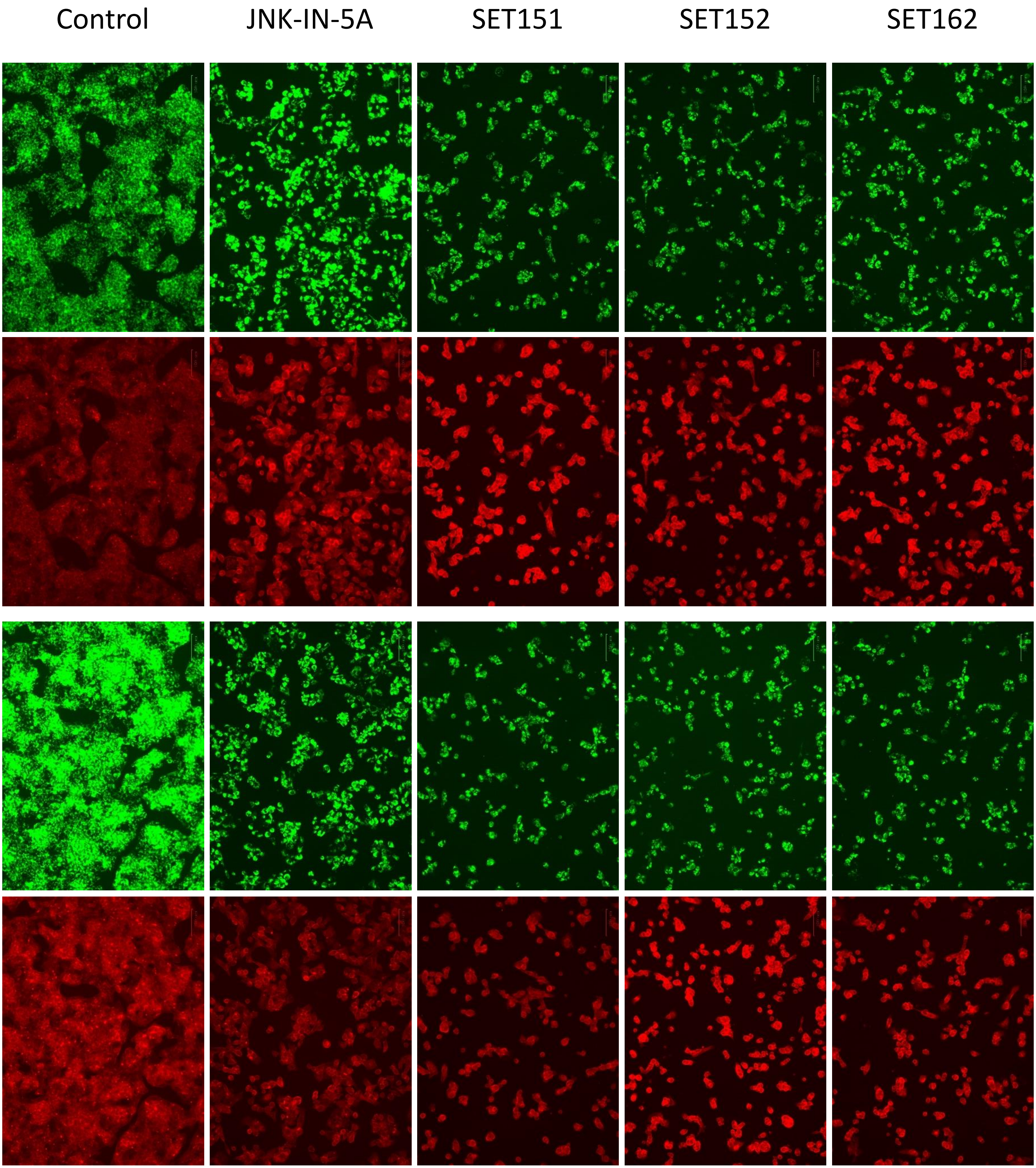

GFP - BODIPY BODIPY™ 493/503
RFP - Phalloidin Alexa Fluor™ 594

Figure 2C

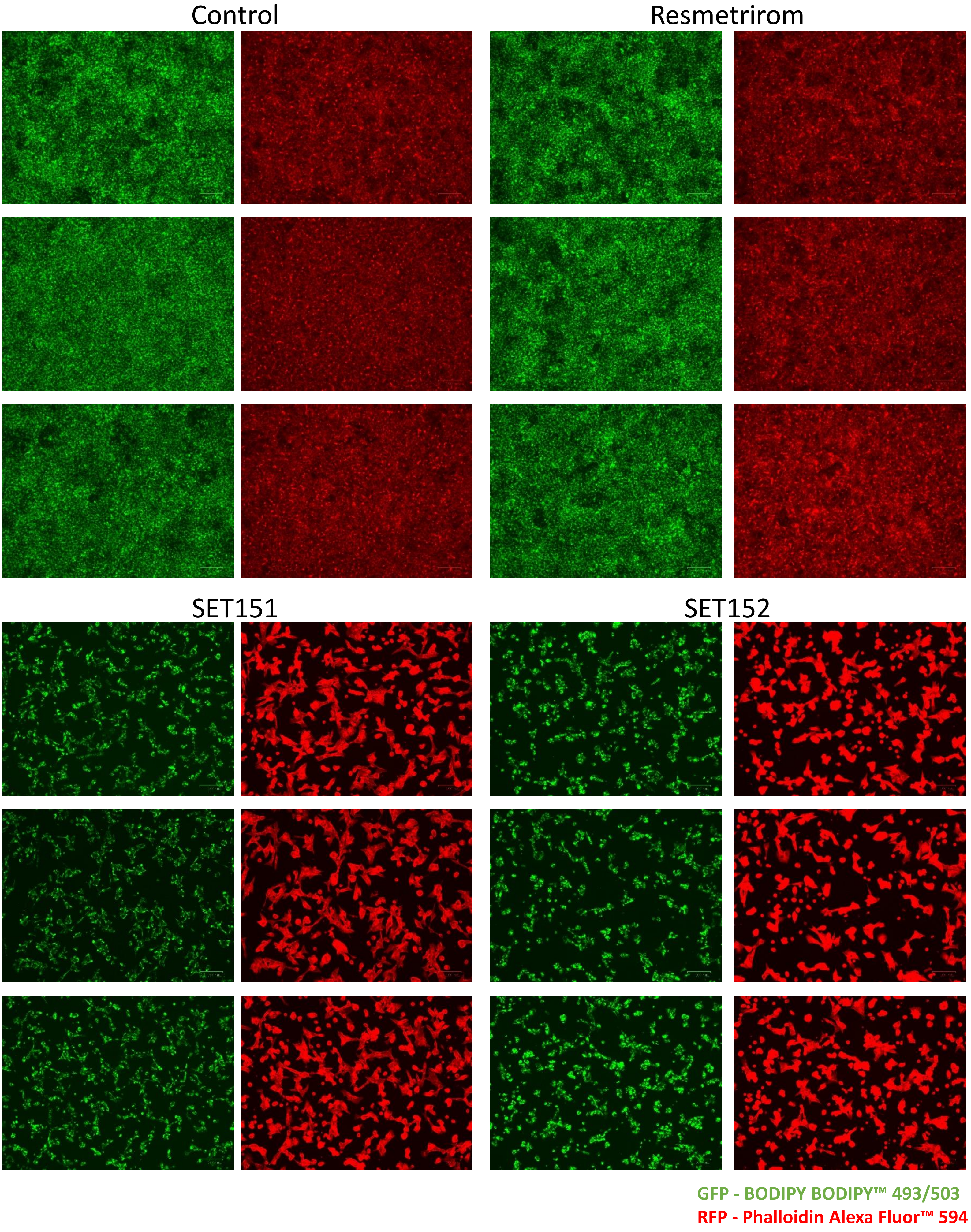
Control
Resmetrirom
SET151
SET152
GFP - BODIPY BODIPY™ 493/503
RFP - Phalloidin Alexa Fluor™ 594

Figure 2C

SET162

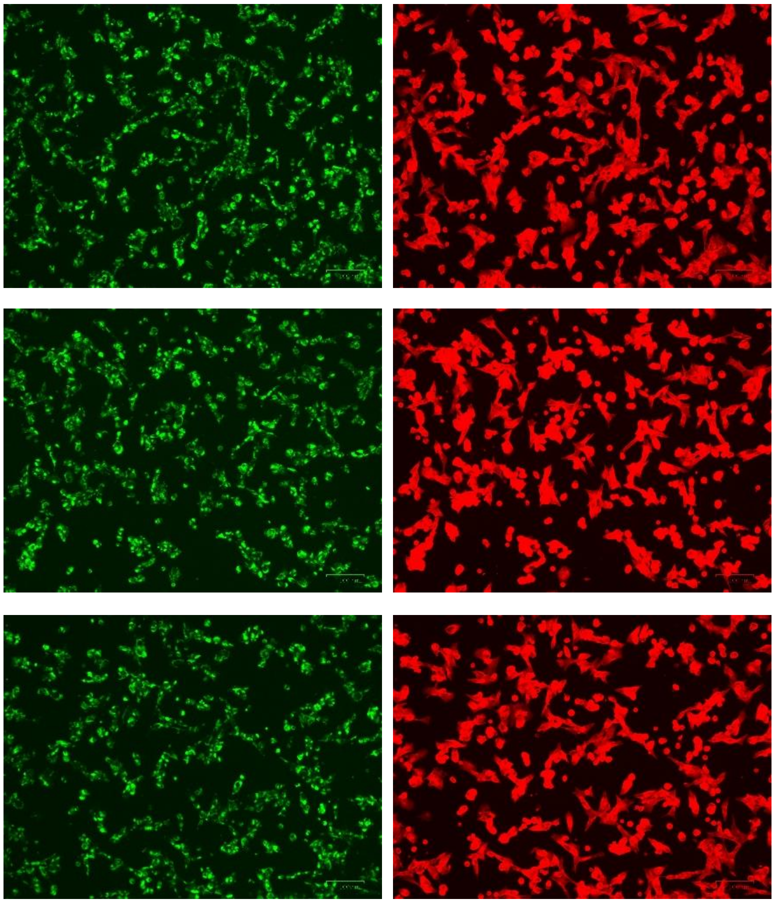

GFP - BODIPY BODIPY™ 493/503
RFP - Phalloidin Alexa Fluor™ 594
